# Strategic distribution of seeds to support diffusion in complex networks

**DOI:** 10.1371/journal.pone.0205130

**Published:** 2018-10-16

**Authors:** Jarosław Jankowski, Marcin Waniek, Aamena Alshamsi, Piotr Bródka, Radosław Michalski

**Affiliations:** 1 Department of Computer Science and Information Technology, West Pomeranian University of Technology, Szczecin, Poland; 2 Masdar Institute, Khalifa University of Science and Technology, Abu Dhabi, United Arab Emirates; 3 The MIT Media Lab, Massachusetts Institute of Technology, Cambridge, MA, United States of America; 4 Department of Computational Intelligence, Faculty of Computer Science and Management, Wrocław University of Science and Technology, Wrocław, Poland; East China Normal University, CHINA

## Abstract

Usually, the launch of the diffusion process is triggered by a few early adopters–i.e., seeds of diffusion. Many studies have assumed that all seeds are activated once to initiate the diffusion process in social networks and therefore are focused on finding optimal ways of choosing these nodes according to a limited budget. Despite the advances in identifying influencing spreaders, the strategy of activating all seeds at the beginning might not be sufficient in accelerating and maximising the coverage of diffusion. Also, it does not capture real scenarios in which marketing campaigns continuously monitor and support the diffusion process by seeding more nodes. More recent studies investigate the possibility of activating additional seeds as the diffusion process goes forward. In this work, we further examine this approach and search for optimal ways of distributing seeds during the diffusion process according to a pre-allocated seeding budget. Theoretically, we show that a universally best solution does not exist, and we prove that finding an optimal distribution of supporting seeds over time for a particular network is an NP-hard problem. Numerically, we evaluate several seeding strategies on different networks regarding maximising the coverage and minimising the spreading time. We find that each network topology has a best strategy given some spreading parameters. Our findings can be crucial in identifying the best strategies for budget allocation in different scenarios such as marketing or political campaigns.

## Introduction

The increasing number of people who use social media has presented a new channel for marketing campaigns. Viral marketing targets potentially influential nodes in social networks to spread the word about certain products or services. However, the optimisation problem of selecting influential nodes as seeds is NP-hard [1]. Due to the computational complexity of the problem, seed selection methods are often based on heuristics. In simple approaches, nodes are selected according to network metrics such as degree or betweenness. In other lines of research, seed selection is based on a greedy approach, [1] which offers reasonable results compared to the exact solution, but unfortunately still has high computational requirements. This is why proposed modifications of the greedy approach are focused on the reduction of the time needed to obtain the seed set [2]. More recent approaches like VoteRank [3] or the community-based selection [4] focus on the information-spreading potential of individual seeds as well as their location in the network in order to select seeds in regions that would not be naturally activated by the spreading process.

Despite the advances in the search for a set of influential nodes in a network, the problem of diffusion is considered stochastic after being triggered by selected influential nodes. This assumes all seeds (influential nodes) are activated at the beginning, then diffusion takes place, and marketing campaigns never interfere with them. In many real cases, however, marketing campaigns actively monitor the diffusion process and disrupt it by activating more seeds to ensure the continuity of adoption of their products or services. Recently, this scenario has been well addressed in many studies. Researchers attempted to use the extra knowledge about ongoing information-spreading processes [5] or explicitly used a scheduling algorithm to allocate seeds over time [6]. Some innovative strategies have been studied, including sequential seeding [7], dynamic rankings [8] and supporting seeding [9]. The empirical results show that the application of additional seeds during the information-spreading process delivers better results than activating all the seeds at the beginning. The reason for the success of continuous seeding is that it utilises natural propagation processes more efficiently and avoids seeding nodes that have a high potential for natural activation.

Following the increasing attention directed toward continuous seeding, new questions arise about when additional seeds should be used and how many of them should be distributed over time, assuming that the campaign must finish in a given period. These questions are very important for marketing campaigns since budgets are allocated in advance for seeding. Hence a decision maker should know when to spend the budget and how to effectively and timely activate seeds that would increase the coverage. In this study, we investigate how the distribution of additional seeds affects the coverage of the network. We address this problem theoretically and numerically to search for optimal distributions of seeds over time in different network topologies. First, we theoretically prove that the optimisation of distribution of seeds over time is NP-hard and a universally best solution does not exist. Due to this finding, we expect to find approximate solutions by examining a number of heuristics for different network topologies hoping to find the best distribution for each class of networks. We use four well-known distributions as heuristics: (1) linear; (2) Gaussian; (3) Geometric; and (4) Decreasing Geometric distributions. We benchmark our heuristics against the standard strategy of seeding nodes that activates all the seeds at the beginning of the process. To simulate the spread of information, we use the independent cascade model [1]. We find that the strategy of distributing seeds over time always beats the baseline strategy. In compliance with our theoretical findings, the best distribution of seeds varies for different network topologies. Our findings can help marketing campaigns better plan for the distribution of seeds over time and hence decide in advance how to spend their allocated budgets.

## Results

### Conceptual framework

**Fig 1:**
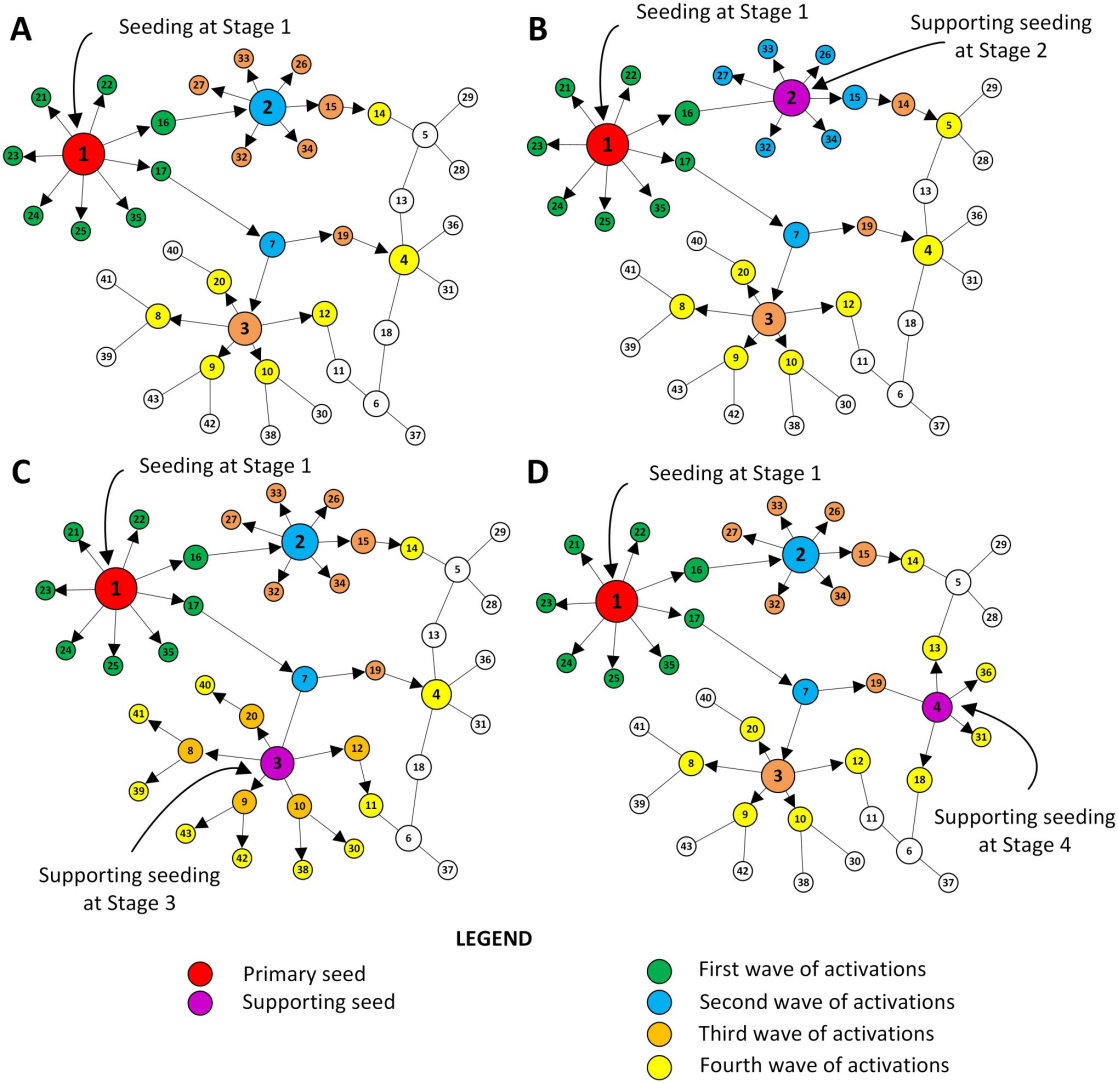
An illustrative example. The figure presents four different variants of the diffusion process consisting of four stages, with propagation probability *PP* = 1 and selecting a node with the highest degree as a seed. Node 1 (marked in red) is selected as the primary seed. Each node’s color corresponds to the stage of activation the node is activated in. Panel A presents the diffusion process without any supporting seeding. The output is 26 activated nodes, covering 60.47% of the network. Panel B shows the process with a single supporting seed that is activated between first and second stages of activations, leading to the total number of 27 activated nodes (coverage of 62.79%). Panel C presents the process in which the supporting seed is activated between second and third stages of activations. As a result, 34 nodes become activated and the coverage increases to 79.07%. Finally, panel D shows the process with a single supporting seed activated between third and fourth stages of activations. As a result, the process ends with 30 active nodes (total coverage of 69.77%).

We begin with a simple illustrative example based on a single primary seed that triggers the spread of information and a single supporting seed that is added after the spread takes place in a network with 43 nodes, presented in Fig 1. Both seeds are selected based on their high degrees, which is one of the selection criteria that we consider in this study. The example illustrates the difference in the total coverage of the network (total number of activated nodes) after adding the supporting seed at different stages of the process. For simplicity, we assume that the propagation probability *PP* is equal to one and that information cascades finish in four stages. Fig 1A illustrates the process of information diffusion without any supporting seeds. We consider this case as a null benchmark. Node 1 is selected as the primary seed (marked with red colour) due to its highest degree, this is true for all considered scenarios. The green nodes are activated at the first stage, the blue nodes are activated at the second stage, the orange nodes are activated at the third stage and the yellow nodes are activated at the fourth stage. As a result, 26 nodes within the network are activated.

Fig 1B shows that the total number of activated nodes becomes 27 when node 2 was selected as a supporting seed after the first stage of activation takes place. Adding an additional seed immediately after diffusion takes place, increases the coverage by only one node in this case. Fig 1C demonstrates the result of delaying the usage of the additional supporting seed for one stage. When node 3 is selected as a supporting seed after the second stage of activation, it increases the coverage significantly to 34 activated nodes. In comparison to the null benchmark where no supporting seeding is added (Fig 1A), node 3 was activated later despite its influence on activating its neighbours while the strategy of supporting seeding activated it faster, which led to maximising the total number of activated nodes. Fig 1D shows that delaying the support until after the third stage of activation and selecting node 4 as a supporting seed results in a lower improvement as only 30 nodes are activated, even though nodes 2 and 3 are activated by the natural diffusion process. This toy example demonstrates not only the important role of supporting seeding in increasing the overall coverage but also the role of choosing the right time to activate supporting seeds in order to maximise the coverage of the network.

Note that while the propagation probability equal to one makes the example presented in Fig 1 more understandable, it also simplifies the problem significantly in comparison to the general setting considered in our work. In particular, it causes the process to be deterministic, shifting focus from selecting stages to add new seeds, to the criteria of selecting the seeds themselves. Since we are able to precisely predict which nodes will become activated at all stages, it is optimal to activate selected seeds at the beginning. Bear in mind, however that in the general version of our problem the propagation probability is usually lower than one, making the process indeterministic. The decision maker may be then inclined to delay the decision of activating a particular node as supporting seed, as it may or may not be activated by the stochastic process. Hence, the decision process in the general case is more complex than might be suggested by the presented example.

The example also illustrates the importance of our assumption that the diffusion process has to end after a certain period. Given the chance, the diffusion process presented in Fig 1 would continue and, in the end, activate the entire network. However, because of the assumed time limit of four stages, the effectiveness of our method of choosing seeds is measured at a particular moment in time. We designed this feature of the process to reflect properties of the situations that we wish to model. For example, in the elections setting, the total coverage of the network is crucial on the election day, while the situation is much less important for the candidate a few days later.

We now move to its formal definition and investigate some of its theoretical properties.

### Modelling supporting seeding

We now present basic network notations, formally define the main problem of this study and analyse some of its theoretical properties.

Let *G* = (*V*, *E*) denotes a network, where *V* denotes the set of nodes and *E* ⊆ *V* × *V* denotes the set of edges. We denote an edge between nodes *v* and *w* by (*v*, *w*). In this work we consider only *undirected* networks, *i*.*e*., networks in which we do not discern between edges (*v*, *w*) and (*w*, *v*). We also assume that networks do not contain self-loops, *i*.*e*., ∀_*v*∈*V*_(*v*, *v*) ∉ *E*. We denote by *N*(*v*) the set of *neighbours* of *v* in *G*, *i*.*e*., *N*(*v*) = {*w* ∈ *V*: (*v*, *w*) ∈ *E*}. Finally, we denote by *d*(*v*) the *degree* (the number of neighbours) of *v* in *G*, *i*.*e*., *d*(*v*) = |*N*(*v*)|.

#### Computational aspects of supporting seeding

First, we formally define the problem of finding an optimal way of supporting seeding.

**Definition 1** (Supporting seeding problem). *The supporting seeding process is defined by the network G* = (*V*, *E*), *the number of primary seeds p, the number of supporting seeds m, the number of supporting seeding stages T, the propagation probability PP, and the strategy of choosing seeds SR*.

*At the beginning of the process p primary seeds are selected using strategy SR. At each of the T* + 1 *activation stages each newly activated node has a chance to activate each of her not active neighbours with propagation probability PP. After each activation stage, with the exception of the last one, more supporting seeds can be added, again using Strategy SR*.

*The goal of the problem is to find the distribution of supporting seeds* ϕ:N→N *among supporting seeding stages such that* ∀_1≤*i*≤*T*_ *ϕ*(*i*) ≥ 0, $\sum_{i=1}^{T}\phi \left(i\right)\leq m$ *and the expected number of activated nodes is maximal*.

In other words, we want to investigate how one should distribute supporting seeds throughout the entire process in order to achieve maximal network coverage. It might seem that at least for simple strategies of choosing the seeds, such as selecting the node with highest degree, there should exist simple observations, such as: *it is always better to activate supporting seeds in the first half of the process*, or *you should never activate supporting seeds near the end of the process*. However, we now show that none of such simple statements can be true in the general case.

**Theorem 1**. *Assume that the probability of activation is PP* = 1, *and that the strategy of choosing seeds SR is choosing nodes with highest degrees to become seeds. For any number of primary seeds* p∈N, *any number of supporting seeds* m∈N, *any number of supporting seeding stages* T∈N, and any temporal distribution of supporting seeds ϕ* there exists a network G, such that ϕ* *is the optimal distribution for* (*G*, *p*, *m*, *T*, *PP*, *SR*). What is more, the difference between the number of activated nodes using ϕ* *and using the next best solution can be arbitrarily high*.

The proofs of all theorems can be found in the supporting materials.

Mainly, the theorem shows that in a general case it is not possible to find *one* universally beneficial rule of distributing supporting seeds for every network structure. The problem is highly dependent on the structure of the network itself. We demonstrate this by presenting a network structure where a large group of nodes can be activated only by choosing a particular distribution of supporting seeds over time. Delaying or advancing the activation of these seeds can dramatically change the final outcome of the process.

Even when focusing on a particular network, it might be computationally infeasible to find the optimal distribution of supporting seeds, as we show in the following theorem.

**Theorem 2**. *Supporting seeding problem is NP-hard*.

In other words, there exist examples of network structures, where there is no polynomial algorithm that finds the optimal distribution of supporting seeds for these structures. However, one might hope that this kind of structures rarely exist in real-life networks. To shed some light on the practical aspects of utilizing supporting seeding we perform experiments on a number of real-life network datasets. Considered strategies of distributing supporting seeds and detailed descriptions of experimental setup are presented in following sections.

### Considered seed distributions

**Fig 2:**
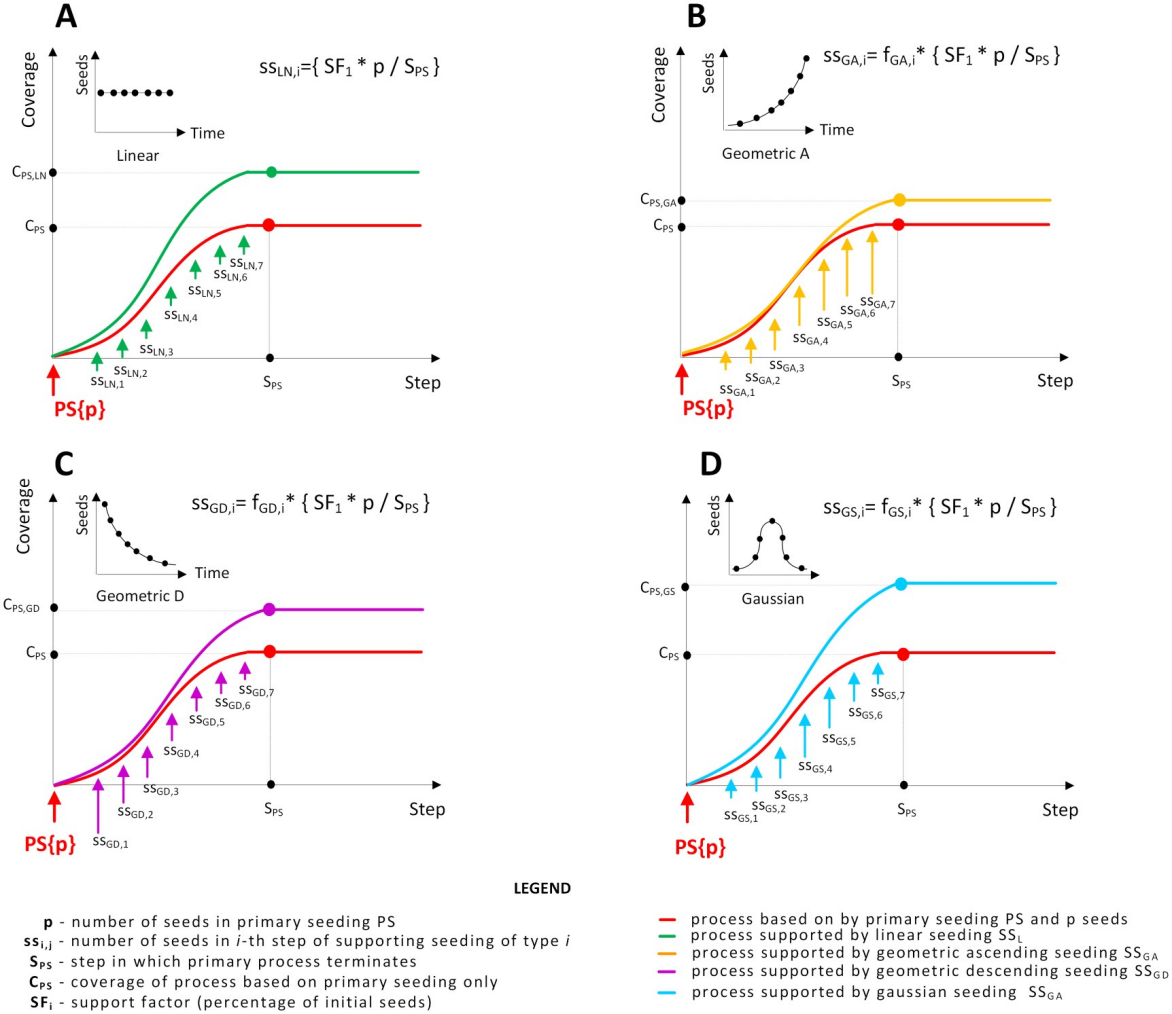
Distributions of supporting seeds. Plots illustrate coverage of the network increasing during the seeding process, while insets illustrate number of supporting seed used in each supporting seeding stage. Each arrow represents a single seeding stage, with red arrow representing primary seeding, and other arrows representing supporting seeding. Size of each arrow corresponds to the number of seeds used in particular seeding stage. Panel (A) presents the linear distribution of supporting seeds, with the same number of supporting seeds used in each stage. Panel (B) presents the ascending geometric distribution of supporting seeds, with low number of seeds used at the beginning of the process and high usage in later stages. Panel (C) presents the descending geometric distribution of supporting seeds, with high number of supporting seeds used at the beginning and significant drop in the later stages. Panel (D) presents the Gaussian distribution of supporting seeds, with maximal intensity in the middle of the process.

We now describe four strategies of distributing supporting seeds among supporting seeding stages considered in our experiments, namely linear distribution, geometric ascending distribution, geometric descending distribution and Gaussian distribution. Scenarios utilizing these distributions are presented in Fig 2.

We can see that different distributions of supporting seeds over time can result in varying total coverage of the network in Fig 2. A linear distribution of supporting seeds (LN) is presented in Fig 2A. In this case, the same number of supporting seeds is used in each of the supporting seeding stages. *C*_*PS*,*LN*_ represents the coverage of the network at the end of the process with linear distribution of supporting seeds, while *C*_*PS*_ represents the coverage of the network without any supporting seeding. The number of supporting seeds activated in round *i* using the linear distribution is computed as follows:

$$\phi_{LN}\left(i\right)=\left\lfloor \frac{m}{T}\right\rfloor .$$

The second distribution is an ascending geometric distribution of supporting seeds (GA) that is presented in Fig 2B. It results in minimal support at the beginning of the process, and the highest impact near the end of the process. The number of supporting seeds in round *i* is determined using the following function:

$$\phi_{GA}\left(i\right)=\lfloor \frac{f_{GA}\left(i\right)}{\sum_{j=1}^{T}f_{GA}\left(j\right)}m\rfloor$$

where

$$f_{GA}\left(i\right)=2^{-(T-i+1)}.$$

The third distribution is geometric (GD) and is presented in Fig 2C, where a high number of supporting seeds are activated at the beginning stages of the process, whereas the number of supporting seeds decreases over time. In our experiments we distribute the seeds proportionally to the geometric distribution where the probability of success in each Bernoulli trial is equal to $\frac{1}{2}$. Hence, the number of supporting seeds activated in round *i* is computed using the following equation:

$$\phi_{GD}\left(i\right)=\lfloor \frac{f_{GD}\left(i\right)}{\sum_{j=1}^{T}f_{GD}\left(j\right)}m\rfloor$$

where

$$f_{GD}\left(i\right)=2^{-i}.$$

The results of using the fourth distribution is shown in Fig 2D, where supporting seeding is following the Gaussian distribution (GS). The highest intensity of the supporting seeding takes place in the middle of the process, with low support is given at the beginning and at the end. To be precise, in our experiments we distribute the seeds proportionally to the normal distribution with standard deviation equal to 1 on (−4, 4) interval, *i*.*e*., the number of supporting seeds activated in round *i* while using the Gaussian distribution is computed using the following function:

$$\phi_{GS}\left(i\right)=\lfloor \frac{f_{GS}\left(i\right)}{\sum_{j=1}^{T}f_{GS}\left(j\right)}m\rfloor$$

where

$$f_{GS}\left(i\right)=e^{-8{\left(\left(2i-1\right)/T-1\right)}^{2}}.$$

We expect that the way of distributing the supporting seeds over time can affect the total coverage of the network in two different ways. The usage of supporting seeds at the beginning of the process may lead to selecting as supporting seeds nodes with high potential of being activated by the natural process, *e.g*., nodes in directed vicinity of primary seeds. This might suggest delaying the supporting seeding until later stages of the process. At the same time however, using supporting seeds too late in the process might also have undesirable effects. Information cascades launched by them cannot get high coverage because the entire process will soon be finished, as a result of a finite time condition.

Next, we move to describing the setup of our experiments in which we compare the coverage of the network using different types of distributions.

### Experimental setup

We now describe the parameters of our experimental setting.

#### Networks

We run our experiments on nine different real-life networks (parameter N), such as scientific collaboration networks. We used the following networks: N1—Condensed matter collaborations 1999 [10], N2—The structure of scientific collaboration networks [11], N3—Power Grid [12], N4—Scholarly Collaboration in Network Science[12], N5—General Relativity and Quantum Cosmology collaboration network [13], N6—DBLP [14], N7—University of Oregon Route Views [13], N8 and N9—Protein interaction network) [15] [16]. The characteristics of these networks are presented in Table 1. Apart from the number of nodes and edges, we also present average values of the following network metrics: degree (DG), closeness (CL), PageRank (PR), eigenvector (EV), clustering coefficient (CC), and betweenness (BT). Moreover, the number of components for networks N1-N9 is equal to 726, 581, 1, 268, 2, 40, 1, 39 and 10 respectively. Fig C in S1 File shows degree distributions for used real networks.

**Table 1:** Specification of used networks.

N	Nodes	Edges	Average values of network measures						Ref
			DG	CL	PR	EV	CC	BT	
N1	16726	47594	2.85	0.00036	0.00006	0.0054	0.6380	33238.58	[10]
N2	8361	15751	1.88	0.00046	0.00013	0.0032	0.4856	13478.94	[11]
N3	4941	6594	1.33	0.05368	0.00020	0.0048	0.0801	44433.29	[17]
N4	1589	2742	1.73	0.00075	0.00068	0.0137	0.6937	251.35	[12]
N5	5242	14496	3.30	0.15145	0.00016	0.0225	0.0109	11468.14	[13]
N6	12591	49743	3.95	0.00989	0.00008	0.0095	0.1166	21217.52	[14]
N7	6474	13895	2.15	0.27657	0.00015	0.0103	0.2522	8754.74	[13]
N8	1706	6207	3.64	0.01038	0.00059	0.0335	0.0012	2946.64	[15]
N9	3133	6726	2.15	0.00379	0.00033	0.0192	0.0658	4917.14	[16]

To model the flow of information in the network we use the Independent Cascade model with propagation probability (parameter PP) between 0.05 and 1.00. The number of nodes selected as primary seeds is determined by parameter PS, and equals *p* = *PS*|*V*|. The number of supporting seeds to be activated during the process is determined by parameter SS, and equals *m* = *SSp*. Both primary and supporting seeds are selected using the same ranking, being it either degree, PageRank, eigenvector or betweenness ranking, and this choice is determined by parameter SR. Finally, the distribution of supporting seeds is determined by parameter SD, and follows one of the distributions presented in the previous section, *i*.*e*., either linear, ascending geometric, descending geometric or Gaussian distribution. Table 2 summarizes all the parameters and presents their possible values.

**Table 2:** Parameters used for simulations.

Symbol	Parameter	Distinct values	Values
N	Network	9	Networks N1-N9 presented in Table 1
PP	Propagation probability	20	0.05, 0.1, 0.15, 0.2 0, 25, 0.3, 0.35, 0.4, 0.45, 0.5, 0.55, 0.6, 0.65, 0.7, 0.75, 0.8, 0.85, 0.9, 0.95, 1.0
PS	Percentage of nodes of the network used as a primary seeds	1	1%
SS	Number of supporting seeds as percentage of number of primary seeds	5	100%, 200%, 300%, 400%, 500%
SR	Ranking method used for seed selection	4	DG—degree, PR—PageRank, EV—eigenvector, BT—betweenness
SD	Supporting seeds distribution	4	LN—linear, GA—ascending geometric distribution, GD—descending geometric distribution, GS—Gaussian distribution

With the parameters described above, the experimental space N x PP x SD x PS x SS x SR contains 14,400 configurations. In order to obtain the number of activation stages determining the number of supporting seeding stages *T*, we first run the experiments from the experimental space N x PP x PS x SR, *i*.*e*., experiments with only primary seeding and without any supporting seeding. We repeat each experiment 100 times to determine the average number of activation stages after which the process without any supporting seeding terminates. We use this value as the number of activation stages in our experiments with supporting seeding. For example, the experiments on network N5, with propagation probability PP = 0.1, and seeds chosen according to degree ranking, the diffusion process without supporting seeding finished on average after 14 simulation steps. Therefore, in experiments with supporting seeding and these parameters we will set the number of activation stages to 14.

### Results of computer simulations

We now present the results of our experiments. For each combination of parameters in the experimental space N x PP x SD x PS x SS x SR described in the previous subsection we perform 100 simulation runs and present average results of coverage. The main goal of the simulations is to compare the performance of supporting seeding using the suggested distributions and quantify the impact of each predefined parameter of the experimental space on the final coverage.

**Fig 3:**
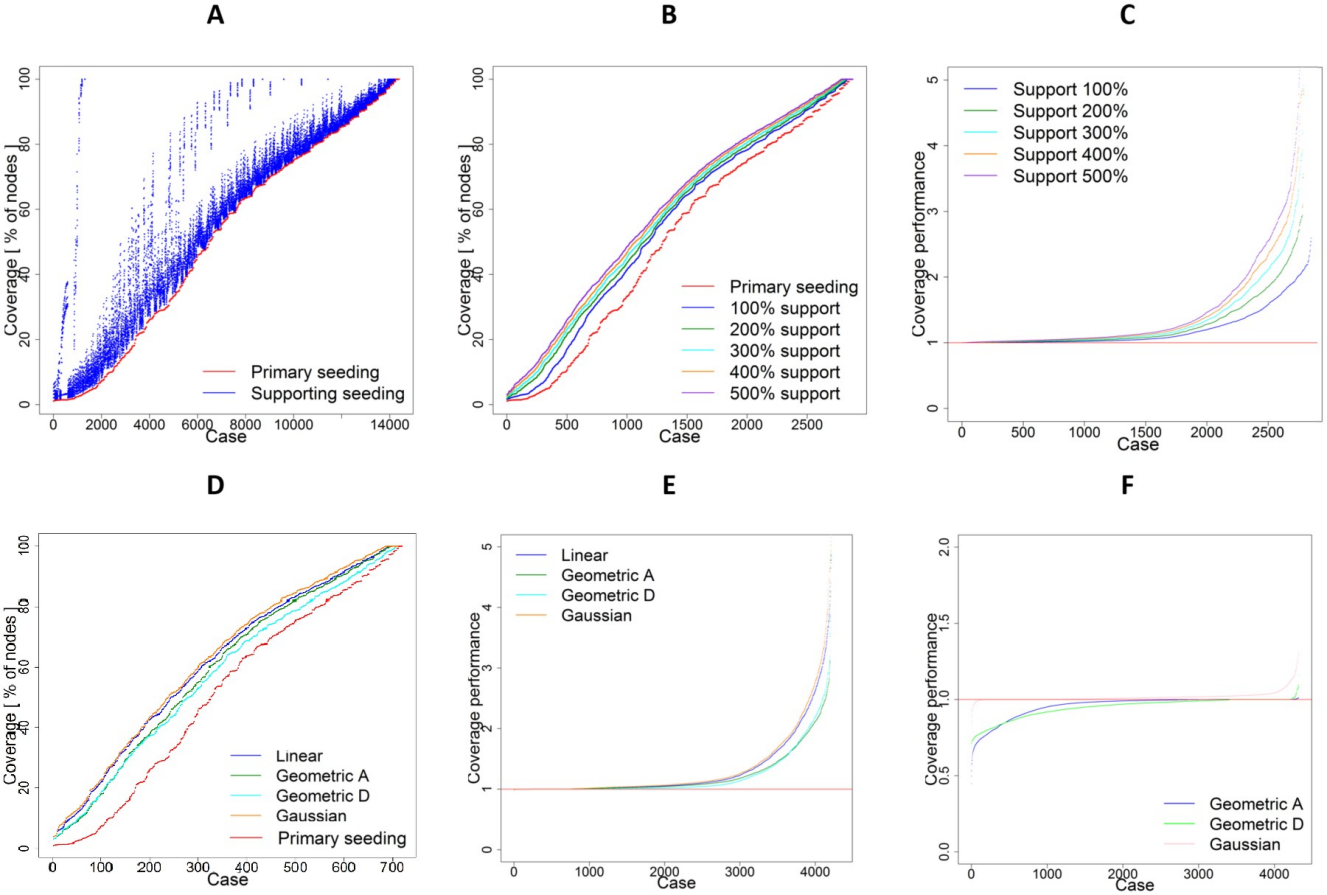
Comparison of results of experiments with and without supporting seeding. (A) Total coverage of a network in experiments with only primary seeding and with supporting seeding presented for every combination of experiment parameters. All averaged results from all runs for all 14400 simulation cases are presented for all possible combinations of parameters listed in the Table 2 and ordered by coverage with primary seeding. (B) Total coverage of a network in experiments with the number of supporting seeds equal to 100%, 200%, 300%, 400%, 500% of primary seeds and in processes with primary seeding only. Average values for 2880 simulation cases for each support are presented and ordered by coverage. (C) Total coverage of a network in experiments with different supporting seeding intensities in relation to the coverage in experiments with primary seeding only. (D) Total coverage of a network in experiments with different distributions of supporting seeds for 500% support and total 720 cases for each used distribution. (E) Total coverage of a network in experiments with different distributions of supporting seeds in relation to the coverage in experiments with primary seeding only. (F) Total coverage in experiments with geometric and Gaussian supporting seeding distributions in relation to experiments with linear distribution.

We start with comparing the aggregate performance of supporting seeding and primary seeding. The coverage of primary seeding is ordered in an increasing order. Fig 3A compares between the average final coverage of the network in two cases: (1) in the presence of only primary seeding and (2) in the presence of both primary and supporting seeding using different combinations of parameters. As it can be seen from the figure, the difference between these two scenarios varies greatly depending on the parametrization of the experiment. Despite these nuanced differences according to selected parameters, supporting seeding always outperform primary seeding which activate all seeds only at the beginning of the process.

Fig 3B illustrates the average total coverage of the network in experiments with only primary seeding, and in experiments with different intensities of supporting seeding (SS parameter) between 100% and 500% of the number of primary seeds for all combinations of parameters presented in the Table 2. Fig 3C presents the ratio between the total coverage of a network for each supporting seeding intensity and the total coverage with primary seeding only. The total coverage in a process with support equal to 100% of primary seeds is on average 1.43 times higher than in a process with only primary seeding. Support intensity of 200% gives on average a 1.84 times higher coverage. Finally, intensities of 300%, 400% and 500% give 1.94 times, 2.04 times and 2.18 times higher coverage respectively.

Now, we discuss the performance of all our strategies of distributing supporting seeds over time. Fig 3D compares the performance of primary seeding and supporting seeding using linear, geometric ascending, geometric descending and Gaussian distributions of supporting seeds. The ratio between results of experiments with each distribution and experiments with primary seeding only is presented in Fig 3E. On average, linear distribution of supporting seeds results in 1.79 times higher coverage than primary seeding only. The ascending geometric distribution delivers 1.67 times better results on average than process with primary seeding only. The descending geometric distribution delivers slightly better results, with an average of 1.7. The best results are achieved with the use of the Gaussian distribution, giving 1.81 times higher coverage one average than in a process with primary seeds only.

To further compare between the performance of different distributions of supporting seeds, Fig 3F shows the fraction between results of linear distribution of supporting seeds and of other distributions. In 94.51% of cases the linear support delivers better results than the ascending geometric distribution, while in 97.62% of cases it is better than descending geometric distribution. The best performing approach based on Gaussian distribution is in 73.19% cases better than the linear distribution, in 76.99% of cases better than ascending geometric and in 78.8% of cases better than descending geometric distribution.

To know whether the differences between distributions in relation to linear distribution are statistically significant, we use Wilcoxon group test and Hodges Lehmann estimator. The linear support compared with ascending geometric support shows H = 1.0748 with statistical significance (p-value < 2.2*e*^−16^). The comparison of linear support versus descending geometric support shows H = 2.2164 and p-value < 2.2*e*^−16^, which confirms that the statistical significance results for linear distribution are better than these for ascending geometric distribution. The difference between linear and Gaussian approach shows H = -0.790129 and confirms with statistical significance (p-value < 2.2*e*^−16^) that the Gaussian distribution of supporting seeds delivers better results.

We now analyse the average number of additionally activated nodes per each supporting seed. For linear support this value is equal to 3.01, *i*.*e*., one supporting seed delivered on average over 3 additional activated nodes. For the ascending geometric distribution, each supporting seed delivers on average 2.53 additional activated nodes, while descending geometric distribution results in 2.17 activated nodes per seed, the lowest number of all considered distributions. The Gaussian distribution achieves the best result with 3.26 activations per supporting seed. Another measure of performance is a coverage increase per supporting seed. One supporting seed results in 0.090% increase of coverage for linear support, 0.076% for ascending geometric support, 0.068% for descending geometric support and 0.096% for Gaussian support.

**Fig 4:**
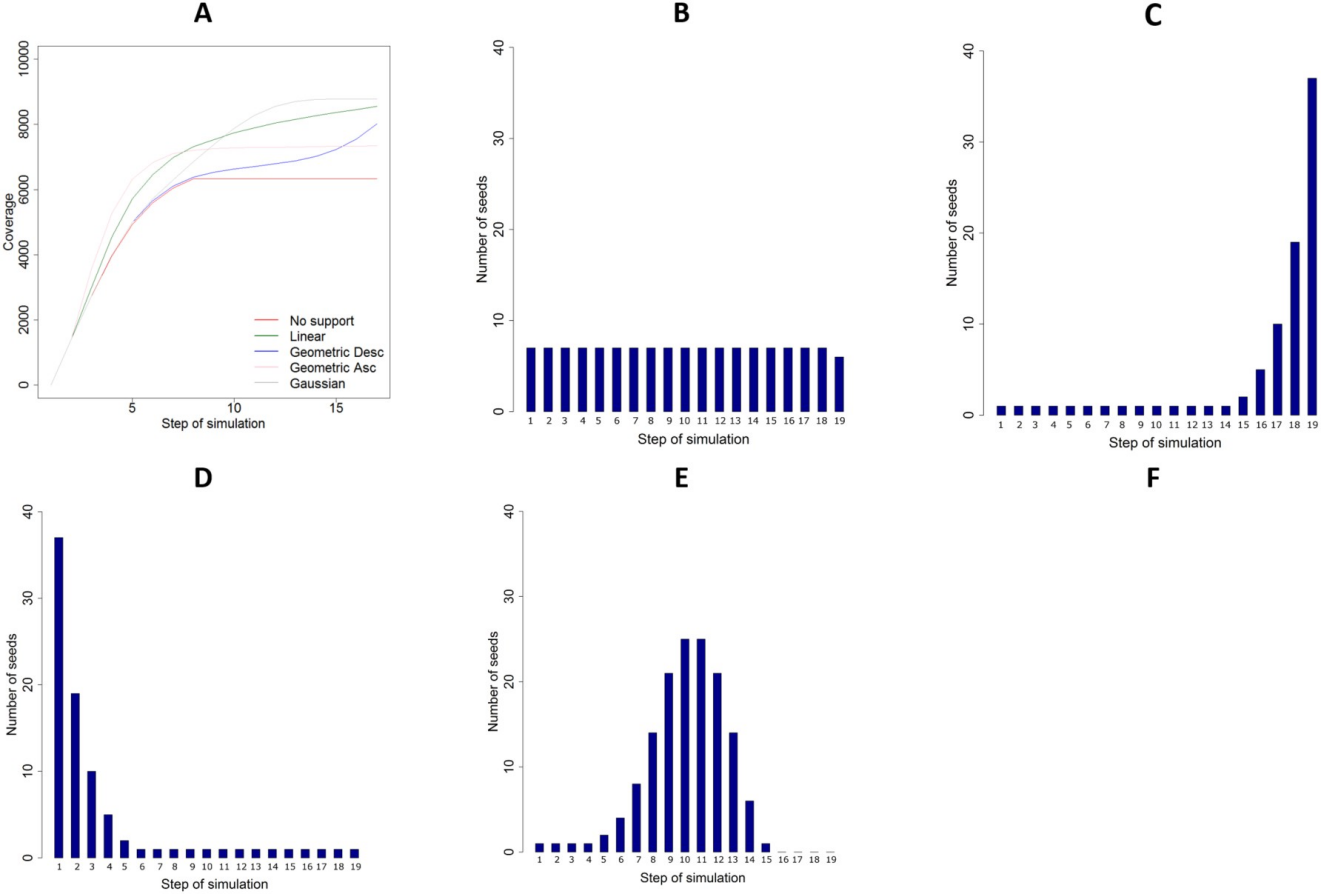
Detailed results of network N1, PP = 0.25 and degree-based selection. (A) Coverage. (B) Average linear distribution of supporting seeds. (C) Average ascending geometric distribution of supporting seeds. (D) Average descending geometric distribution of supporting seeds. (E) Average Gaussian distribution of supporting seeds.

So far we presented aggregated results of various simulations. Next, we present results of using different distributions of seeds over time in one type of networks. Fig 4 presents results of experiments on network N1, with the propagation probability PP = 0.25 and degree based selection of seeds. Fig 4A shows average coverage of N1. The highest increase of coverage is observed for Gaussian distribution of supporting seeds. The average number of supporting seeds in each round for each distribution is showed in Fig 4B–4E.

**Fig 5:**
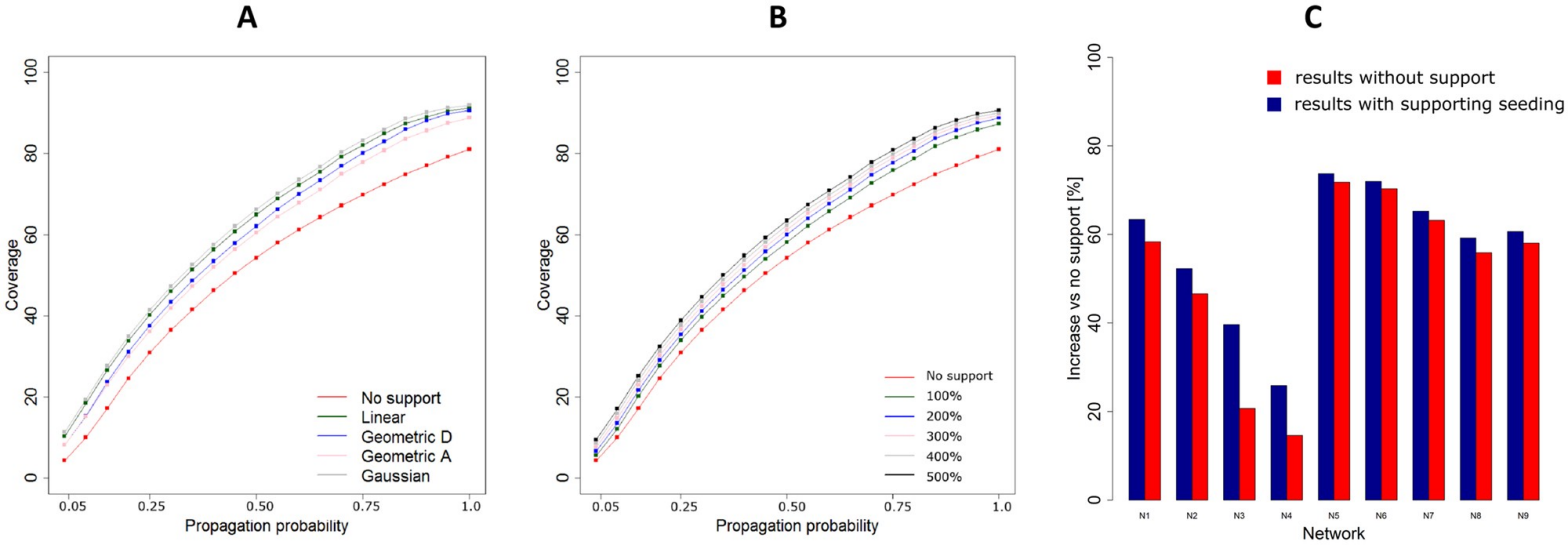
Comparison between coverage in supported and not supported process. (A) Total coverage in supported and not supported processes for different distributions of supporting seeds as a function of propagation probability. (B) Total coverage for different support intensities as a function of propagation probability. (C) Average results for all networks in experiments with and without supporting seeding.

We now analyse how the average performance of supporting seeding varies while using different values of propagation probabilities (parameter PP), the key characteristic of information-spreading process in experiments. Fig 5A shows the coverage of primary seeding and each distribution of supporting seeds using different values of propagation probability. Fig 5B shows the relationship between different propagation probabilities and different total number of supporting seeds.

Finally, we found that the performance of supporting seeding is highly dependent on characteristics of the network itself. The average coverage for each considered network in experiments with and without supporting seeding are presented in Fig 5C. Best results are achieved for networks N3 and N4, characterised by relatively low average degree (see Table 1). This again supports our theoretical findings.

Detailed numerical average results for all combinations parameters are presented in the Table 3. The Gaussian distribution has the best average results in many cases with 1.97 times higher coverage than the process with primary seeding only. It is slightly better than linear distribution of supporting seeds. The Gaussian support delivers 9.23% better results than support with ascending geometric distribution and 7.74% better than support with descending geometric distribution. For all considered distributions a statistical significance with p-value < 2*e*^−16^ was observed.

**Table 3:** Average results for real networks N1-N9 for each used parameter. Each result indicates the size of coverage in the process with supporting seeding, in comparison to the process with only primary seeding.

Parameter	Value	Average results				p-value			
		Linear	GeomA	GeomD	Gaussian	Linear	GeomA	GeomD	Gaussian
**PP**	0.05	2.20	1.81	1.83	2.39	2.00*e*^−16^	3.08*e*^−12^	1.98*e*^−12^	< 2*e*^−16^
	0.10	1.97	1.66	1.67	2.09	3.96*e*^−14^	1.82*e*^−10^	1.25*e*^−10^	9.45*e*^−16^
	0.15	1.76	1.56	1.56	1.83	1.25*e*^−11^	1.96*e*^−9^	2.13*e*^−9^	1.65*e*^−12^
	0.20	1.61	1.44	1.44	1.66	5.71*e*^−10^	2.68*e*^−8^	3.07*e*^−8^	1.51*e*^−10^
	0.25	1.58	1.43	1.43	1.62	1.10*e*^−9^	3.74*e*^−8^	3.73*e*^−8^	4.66*e*^−10^
	0.30	1.59	1.45	1.45	1.62	9.74*e*^−10^	2.50*e*^−8^	2.40*e*^−8^	4.73*e*^−10^
	0.35	1.61	1.48	1.48	1.63	6.11*e*^−10^	1.31*e*^−8^	1.14*e*^−8^	3.49*e*^−10^
	0.40	1.62	1.50	1.51	1.64	4.92*e*^−10^	8.07*e*^−9^	6.83*e*^−9^	2.93*e*^−10^
	0.45	1.66	1.56	1.57	1.70	1.50*e*^−10^	2.12*e*^−9^	1.66*e*^−9^	5.82*e*^−11^
	0.50	1.67	1.58	1.58	1.70	1.29*e*^−10^	1.32*e*^−9^	1.07*e*^−9^	5.45*e*^−11^
	0.55	1.67	1.60	1.60	1.70	1.20*e*^−10^	7.36*e*^−10^	7.86*e*^−10^	5.52*e*^−11^
	0.60	1.68	1.62	1.61	1.70	1.13*e*^−10^	4.86*e*^−10^	5.97*e*^−10^	5.37*e*^−11^
	0.65	1.72	1.64	1.64	1.73	4.08*e*^−11^	2.75*e*^−10^	2.58*e*^−10^	2.89*e*^−11^
	0.70	2.12	1.85	2.07	2.05	3.38*e*^−16^	9.88*e*^−13^	1.91*e*^−15^	2.79*e*^−15^
	0.75	2.17	2.02	2.12	2.14	< 2*e*^−16^	8.32*e*^−15^	3.13*e*^−16^	< 2*e*^−16^
	0.80	2.35	2.16	2.28	2.28	< 2*e*^−16^	< 2*e*^−16^	< 2*e*^−16^	< 2*e*^−16^
	0.85	2.55	2.43	2.49	2.56	< 2*e*^−16^	< 2*e*^−16^	< 2*e*^−16^	< 2*e*^−16^
	0.90	2.52	2.52	2.49	2.53	< 2*e*^−16^	< 2*e*^−16^	< 2*e*^−16^	< 2*e*^−16^
	0.95	2.48	2.47	2.45	2.48	< 2*e*^−16^	< 2*e*^−16^	< 2*e*^−16^	< 2*e*^−16^
	1.00	2.34	2.33	2.33	2.35	< 2*e*^−16^	< 2*e*^−16^	< 2*e*^−16^	< 2*e*^−16^
**Network**	N1	1.26	1.18	1.14	1.29	4.78*e*^−15^	2.45*e*^−13^	1.10*e*^−12^	1.25*e*^−15^
	N3	1.38	1.26	1.22	1.43	< 2*e*^−16^	4.28*e*^−15^	2.73*e*^−14^	< 2*e*^−16^
	N3	4.69	4.01	4.32	4.70	< 2*e*^−16^	< 2*e*^−16^	< 2*e*^−16^	< 2*e*^−16^
	N4	4.54	4.35	4.41	4.59	< 2*e*^−16^	< 2*e*^−16^	< 2*e*^−16^	< 2*e*^−16^
	N5	1.12	1.08	1.07	1.13	3.72*e*^−12^	2.10*e*^−11^	3.40*e*^−11^	1.73*e*^−12^
	N6	1.06	1.04	1.02	1.07	4.98*e*^−11^	9.69*e*^−11^	1.92*e*^−10^	2.49*e*^−11^
	N7	1.09	1.06	1.06	1.11	1.05*e*^−11^	3.71*e*^−11^	4.88*e*^−11^	4.38*e*^−12^
	N8	1.17	1.13	1.10	1.20	3.01*e*^−13^	4.00*e*^−12^	6.81*e*^−12^	7.09*e*^−14^
	N9	1.18	1.13	1.11	1.21	2.30*e*^−13^	2.56*e*^−12^	6.12*e*^−12^	5.33*e*^−14^
**Strategy**	D	1.33	1.24	1.21	1.37	< 2*e*^−16^	< 2*e*^−16^	< 2*e*^−16^	< 2*e*^−16^
	PR	1.46	1.33	1.33	1.51	< 2*e*^−16^	< 2*e*^−16^	< 2*e*^−16^	< 2*e*^−16^
	EV	3.59	3.38	3.52	3.58	< 2*e*^−16^	< 2*e*^−16^	< 2*e*^−16^	< 2*e*^−16^
	BT	1.39	1.27	1.26	1.43	< 2*e*^−16^	< 2*e*^−16^	< 2*e*^−16^	< 2*e*^−16^
**Support**	100%	1.45	1.40	1.42	1.47	< 2*e*^−16^	< 2*e*^−16^	< 2*e*^−16^	< 2*e*^−16^
	200%	1.87	1.77	1.79	1.89	< 2*e*^−16^	< 2*e*^−16^	< 2*e*^−16^	< 2*e*^−16^
	300%	2.00	1.86	1.88	2.03	< 2*e*^−16^	< 2*e*^−16^	< 2*e*^−16^	< 2*e*^−16^
	400%	2.11	1.93	1.98	2.15	< 2*e*^−16^	< 2*e*^−16^	< 2*e*^−16^	< 2*e*^−16^
	500%	2.27	2.06	2.08	2.32	< 2*e*^−16^	< 2*e*^−16^	< 2*e*^−16^	< 2*e*^−16^

In general, supporting seeding delivers best relative increase of coverage in experiments with the lowest used propagation probability PP = 0.05. The Gaussian support delivers 8.62% better results than linear for propagation probability PP = 0.05. Performance is dropping till 2.25% increase for PP = 0.25. Results from Gaussian distribution compared with geometric descending and ascending showed highest increase of performance of Gaussian approach for PP = 0.10 with 30.53% increase for descending and 31.69 for ascending geometric distribution. The lowest increase was observed for PP = 0.05 with a 7.74% and a 9.24% increase respectively. In terms of used networks, supporting seeding delivered best results for networks N3 and N4 with more than 4 times increase for all used algorithms, when compared to information spreading process without any support. The lowest increase was observed for network N6 for all used distributions of supporting seeds. Performance was related to seeds selection strategy. The best performance of supporting seeding was observed when the selection was based on eigenvector, and was more than 3 times higher when compared to the information-spreading process without support. Support equal to initial seeding performance was similar to all used strategies. The highest differences were observed for intensity of support five times higher than initial seeding. Gaussian support delivered highest 2.32 times increase, 2.27 for linear support.

While the emphasis of our research has been put on real networks, we also performed experiments using synthetic networks to search for strategies that could be generalized to a class of networks: Barabási–Albert model (BA), Watts-Strogatz (WS) model and Erdos-Renyi model (ER). Experiments were based on the same propagation probabilities PP, seed selection strategies (Degree, Page Rank, Eigenvector, Betweenness) and support intensities (100%, 200%, 300%, 400%, 500%) as for the real networks. Synthetic networks with 5,000 nodes were generated according to Barabási–Albert model (BA), Watts-Strogatz (WS) model and Erdos-Renyi model (ER). For Barabási–Albert model the number of edges added with each node was 2, while the size of initial clique was 2. For Watts-Strogatz model the expected degree of a node was 4, while the rewiring probability was set to $\frac{5}{100}$. For Erdos-Renyi model the probability of connecting every two edges was set to $\frac{1}{1000}$, hence the expected degree is almost 5. Fig D in S1 File shows degree distributions for used synthetic networks.

Results based on the networks following BA model are presented in Table A in S1 File. Analysis based on propagation probabilities PP shows that in general the best results were achieved for descending geometric distribution of additional seeds over the time. Similar results, especially for low probabilities were obtained for Gaussian distribution of seeds. Slightly worse results were obtained for ascending geometrical distribution. The worst results were achieved by the linear support and same number of seeds used in each step. In terms of seed selection strategies, selection based on Page Rank provides the best results for every distribution.

Results from simulations performed on networks following WS model are presented in Table B in S1 File. Analysis for propagation probabilities PP shows that for PP lower than 0.5 results were the best for linear support. For higher propagation probabilities results were similar for all seed distribution strategies. Linear support was the best for Page Rank and Eigenvector based seed selection while ascending geometric distribution was the best for Degree and Betweenness. Total analysis for supporting seeding intensity shows that for all intensities linear support delivered the best results.

Simulations based on ER network model are presented in Table C in S1 File. For propagation probabilities lower than 0.2 best results were obtained for ascending geometrical distribution of seeds. For propagation probabilities higher than 0.2 and lower than 0.65 the best results were achieved for descending geometrical distribution. For higher propagation probabilities similar results were achieved for all distributions of additional seeds. For all seeds selection strategies the best results were achieved for descending geometric distribution. From the perspective of supporting seeding intensity, results for ascending and descending geometric distribution of seeds are similar.

Our further analysis of randomly generated network is focused on how network characteristics, such as average degree, closeness or betweenness, affect the performance of supporting seeding. The results are presented in the Figs E-G in S1 File. The impact of network characteristics turns out to be more significant for higher propagation probabilities. Networks generated using all three models show similar tendencies in terms of how coverage depends on characteristics’ values. In general, the coverage tends to increase with average degree, closeness, eigenvector and size of second order neighbourhood, while it tends to decrease with average betweenness. Furthermore, we compared the performance of different distributions on networks that are generated using one of the three models while controlling for network characteristics and propagation probabilities. Interestingly, we found that although the differences in the performance of each two distributions are small in each network type although they are statistically significant. More detailed descriptions and interpretations of the results can be found in the Figs E-G in S1 File.

## Conclusion

The increasingly important role of social media in marketing strategies requires new analytical and decision supported solutions. Most of the earlier studies related to viral marketing are focused on seed selection and initialisation of the information diffusion process, without considering additional support. At the same time, real-life marketing campaigns are based on continuous monitoring of performance, using an additional budget to boost campaign dynamics and coverage. However, the budget assigned to supporting campaigns can be allocated according to different strategies. One possible strategy is to assign the same number of supporting seeds at each stage, while another strategy can add more supporting seeds at the beginning or close to the end of a campaign. Spending the additional budget at the beginning of the campaign may result in activating nodes that would be reached anyway by the natural diffusion processes, while postponing supporting seeding to the end of the process enables activating nodes which are difficult to reach with the natural processes, but it might be too late to fully exploit the potential of activating these nodes in initiating new information cascades.

We theoretically investigated whether it would be possible to find one universally useful strategy of distributing supporting seeds for any network structure and we found that the best strategy of distributing supporting seeds is dependent on network structures. However, finding the optimal distribution for a given network structure is NP-hard. Therefore, we explore the problem numerically for a number of real networks and use different combinations of parameters: propagation probabilities, total number of supporting seeds, seed selection criterion, and supporting seeds distributions.

By using different distributions of supporting seeds over time, we show that the performance of different ways of distributing additional seeding is highly dependent on the selected parameters. For many cases, we obtain the best results using the Gaussian distribution, with lower usage of additional seeds at the beginning and the end of the process. The distribution avoids seeding nodes with the potential to be activated on their own and gives seeds enough time to explore new nodes.

Several extensions of the proposed approach can be planned for future work. Other distributions of supporting seeding can be explored on different networks. Another possibility is considering supporting seeding with diffusion models other than independent cascade, e.g. the linear threshold model. New directions can be based on the other assumptions e.g., conditional support and the use of information related to dynamics of spreading processes, detection of optimal points to provide support or prediction of time when the process dynamics drops.

## Supporting information

S1 File Supporting information file with proofs, additional analysis, Figs A-G, Tables A-C and detailed statistics. (PDF)

S1 Data Data file with used networks and detailed results. (ZIP)

## References

[1] Kempe D, Kleinberg J, Tardos É. Maximizing the spread of influence through a social network. In: Proceedings of the ninth ACM SIGKDD international conference on Knowledge discovery and data mining. ACM; 2003. p. 137–146.

[2] Michalski R, Kazienko P. Maximizing social influence in real-world networks—the state of the art and current challenges In: Propagation Phenomena in Real World Networks. Springer; 2015 p. 329–359.

[3] Zhang JX, Chen DB, Dong Q, Zhao ZD. Identifying a set of influential spreaders in complex networks. Scientific reports. 2016;6:27823 10.1038/srep27823 27296252 PMC4906276

[4] He JL, Fu Y, Chen DB. A novel top-k strategy for influence maximization in complex networks with community structure. PloS one. 2015;10(12):e0145283 10.1371/journal.pone.0145283 26682706 PMC4689492

[5] Seeman L, Singer Y. Adaptive seeding in social networks. In: Foundations of Computer Science (FOCS), 2013 IEEE 54th Annual Symposium on. IEEE; 2013. p. 459–468.

[6] Granell C, Gómez S, Arenas A. Competing spreading processes on multiplex networks: awareness and epidemics. Physical review E. 2014;90(1):012808 10.1103/PhysRevE.90.012808 25122343

[7] Jankowski J, Bródka P, Kazienko P, Szymanski BK, Michalski R, Kajdanowicz T. Balancing speed and coverage by sequential seeding in complex networks. Scientific reports. 2017;7(1):891 10.1038/s41598-017-00937-8 28420880 PMC5429852

[8] Jankowski J. Dynamic rankings for seed selection in complex networks: Balancing costs and coverage. Entropy. 2017;19(4):170 10.3390/e19040170

[9] Jankowski J, Michalski R. Increasing coverage of information spreading in social networks with supporting seeding. In: International Conference on Data Mining and Big Data. Springer; 2017. p. 209–218.

[10] Newman ME. Scientific collaboration networks. I. Network construction and fundamental results. Physical review E. 2001;64(1):016131 10.1103/PhysRevE.64.016131 11461355

[11] Newman ME. The structure of scientific collaboration networks. Proceedings of the national academy of sciences. 2001;98(2):404–409. 10.1073/pnas.98.2.404 PMC14598 11149952

[12] Newman ME. Finding community structure in networks using the eigenvectors of matrices. Physical review E. 2006;74(3):036104 10.1103/PhysRevE.74.036104 17025705

[13] Leskovec J, Kleinberg J, Faloutsos C. Graph evolution: Densification and shrinking diameters. ACM Transactions on Knowledge Discovery from Data (TKDD). 2007;1(1):2 10.1145/1217299.1217301

[14] Ley M. The DBLP computer science bibliography: Evolution, research issues, perspectives. In: International symposium on string processing and information retrieval. Springer; 2002. p. 1–10.

[15] Stelzl U, Worm U, Lalowski M, Haenig C, Brembeck FH, Goehler H, et al A human protein-protein interaction network: a resource for annotating the proteome. Cell. 2005;122(6):957–968. 10.1016/j.cell.2005.08.029 16169070

[16] Rual JF, Venkatesan K, Hao T, Hirozane-Kishikawa T, Dricot A, Li N, et al Towards a proteome-scale map of the human protein–protein interaction network. Nature. 2005;437(7062):1173 10.1038/nature04209 16189514

[17] Watts DJ, Strogatz SH. Collective dynamics of ‘small-world’ networks. nature. 1998;393(6684):440 10.1038/30918 9623998

