# Supplementary material for: Strategic distribution of seeds to support diffusion in complex networks
Source: PLoS One. 2018 Oct 16;13(10):e0205130. doi: 10.1371/journal.pone.0205130 (PMC6191084; doi:10.1371/journal.pone.0205130)
Supplement: S1 File — (PDF) [file pone.0205130.s001.pdf]

Supporting information for: Strategic Distribution of Seeds to Support Diffusion in Complex Networks

Jarosław Jankowski, Marcin Waniek, Aamena Alshamsi, Piotr Bródka, Radosław Michalski

Proof of Theorem 1

Let $x \in \mathbb{N}$ such that $x > m$ be the parameter controlling the difference between then number of activated nodes in the optimal and in the second best solution.

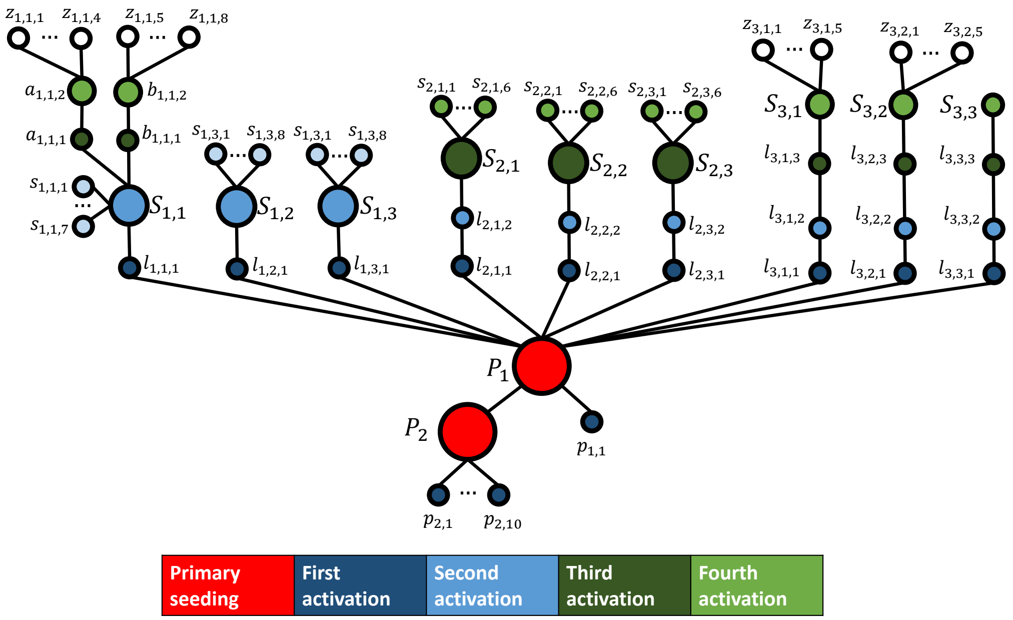

Fig A. Example of a network constructed in the proof of Theorem 1, constructed for $p = 2$, $m = 3$, $T = 3$, $x = 4$, and distribution $\phi^* =< 1, 0, 2 >$, *i.e.*, distribution that activates one supporting seed in stage 1 and two supporting seeds in stage 3. Nodes that get activated in a process without any supporting seeding are colored according to their activation stage. Size of a node corresponds to its degree.

We will now construct the network G such that a given distribution ϕ^* is the optimal distribution of supporting seeds in it. Example of such network is presented in Figure A:

- **The set of nodes:**
  - We create p nodes $P_1, \ldots, P_p$ (these will be the primary seeds).
  - For node P_1 we create $\alpha = \max(0, x - (m-2)T - p + 2)$ nodes $p_{1,1}, \ldots, p_{1,\alpha}$ (the sole purpose of these nodes will be providing P_1 with high degree).

- For every other node P_i such that $i > 1$ we create $x + 2T$ nodes $p_{i,1}, \ldots, p_{i,x+2T}$ (the sole purpose of these nodes will be providing P_i with high degree).
- For every $i = 1, \ldots, T$ and every $j = 1, \ldots, m$ we create:
  * a node $S_{i,j}$ (because of their degree these will be potential supporting seeds);
  * i nodes $l_{i,j,1}, \ldots, l_{i,j,i}$ (these will be the nodes on the path between P_1 and $S_{i,j}$);
  * if $i < T$ we create β nodes $s_{i,j,1}, \ldots, s_{i,j,\beta}$, where $\beta = x + 2(T - i) - 1$ if $j \leq \phi^*(i)$ and $\beta = x + 2(T - i)$ otherwise (the sole purpose of these nodes will be providing $S_{i,j}$ with high degree);
  * if $i < T$ and $j \leq \phi^*(i)$ we also create $2x$ nodes $z_{i,j,1}, \ldots, z_{i,j,2x}$ (these are the nodes that will get activated only if enough supporting seeds get activated in stage i), as well as $T - i$ nodes $a_{i,j,1}, \ldots, a_{i,j,T-i}$ and $T - i$ nodes $b_{i,j,1}, \ldots, b_{i,j,T-i}$ (these will be the nodes at the path between $S_{i,j}$ and nodes $z_{i,j,k}$);
  * if $i = T$ and $j \leq \phi^*(i)$ we also create $x + 1$ nodes $z_{i,j,1}, \ldots, z_{i,j,x+1}$ (these are the nodes that will get activated only if enough supporting seeds get activated in stage i).

- **The set of edges:**
  - For every node P_i such that $i > 1$ we create an edge (P_i, P_1).
  - For every node $p_{i,j}$ we create an edge $(p_{i,j}, P_i)$.
  - For every pair of nodes $l_{i,j,k}$ and $l_{i,j,k+1}$ we create an edge $(l_{i,j,k}, l_{i,j,k+1})$. For every node $S_{i,j}$ we also create edges $(S_{i,j}, l_{i,j,i})$ and $(l_{i,j,1}, P_1)$. In other words, we create a path between $S_{i,j}$ and P_1, running through the nodes $l_{i,j,k}$.
  - For every node $s_{i,j,k}$ we create an edge $(s_{i,j,k}, S_{i,j})$.
  - For every pair of nodes $a_{i,j,k}$ and $a_{i,j,k+1}$ we create an edge $(a_{i,j,k}, a_{i,j,k+1})$. For every node $a_{i,j,1}$ we also create the edge $(S_{i,j}, a_{i,j,1})$. In other words, we create a path consisting of nodes $a_{i,j,k}$ starting at node $S_{i,j}$.
  - For every pair of nodes $b_{i,j,k}$ and $b_{i,j,k+1}$ we create an edge $(b_{i,j,k}, b_{i,j,k+1})$. For every node $b_{i,j,1}$ we also create the edge $(S_{i,j}, b_{i,j,1})$. In other words, we create a path consisting of nodes $b_{i,j,k}$ starting at node $S_{i,j}$.
  - For every node $z_{i,j,k}$ such that $i < T$ and $k \leq x$ we create an edge $(z_{i,j,k}, a_{i,j,T-i})$.
  - For every node $z_{i,j,k}$ such that $i < T$ and $k > x$ we create an edge $(z_{i,j,k}, b_{i,j,T-i})$.
  - For every node $z_{i,j,k}$ such that $i = T$ we create an edge $(z_{i,j,k}, S_{i,j})$.

Now, let us analyze the degrees of nodes of the network as, due to the chosen strategy of seeding, they determine which nodes become seeds:

- We have $d(P_i) > x + 2T$. This is true for P_1 because it is connected with Tm nodes $l_{i,j,1}$, as well as $p - 1$ nodes P_i and $\alpha = \max(0, x - (m-2)T - p + 2)$ nodes $p_{1,i}$ (hence we have that $d(P_1) = \max(Tm + p - 1, x + 2T + 1)$). This is also true for any P_i with $i > 1$ because it is connected to P_1, as well as $x + 2T$ nodes $p_{i,j}$.
- If $i < T$ and $j \leq \phi^*(i)$ we have $d(S_{i,j}) = x + 2(T - i) + 2$ because it is connected to $x + 2(T - i) - 1$ nodes $s_{i,j,k}$, as well as nodes $a_{i,j,1}$,$b_{i,j,1}$ and $l_{i,j,i}$.

- If $i < T$ and $j > \phi^*(i)$ we have $d(S_{i,j}) = x + 2(T - i) + 1$ because it is connected to $x + 2(T - i)$ nodes $s_{i,j,k}$ and the node $l_{i,j,i}$.
- if $i = T$ and $j \leq \phi^*(i)$ we have $d(S_{i,j}) = x + 2$ because it is connected to $x + 1$ nodes $z_{i,j,1}, \dots, z_{i,j,x+1}$ and the node $l_{i,j,i}$.
- We have $d(a_{i,j,T-i}) = x + 1$ because it is only connected to x nodes $z_{i,j,1}, \dots, z_{i,j,x}$ and either node $a_{i,j,T-i-1}$ or node $S_{i,j}$ (if $i = T - 1$).
- We have $d(b_{i,j,T-i}) = x + 1$ because it is only connected to x nodes $z_{i,j,x+1}, \dots, z_{i,j,2x}$ and either node $b_{i,j,T-i-1}$ or node $S_{i,j}$ (if $i = T - 1$).
- Finally, for all other nodes $v \in V$ we have $d(v) \leq 2$.

In what follows, let P denote the set of all nodes P_i, let S denote the set of all nodes $S_{i,j}$, and let Z denote the set of all nodes $z_{i,j,k}$. First, notice that p nodes with highest degrees in the network are nodes in P, therefore they will be chosen as the primary seeds by the strategy SR. Second, notice that for any node $S_{i.j}$ such that $i < T$ and any node $v \in V \setminus (P \cup S)$ we have that $d(S_i) > d(v)$. Therefore, if during any supporting seeding stage there are enough inactive nodes from $S_{i,j}$ such that $i < T$, only they can be chosen as supporting seeds.

Now, let us analyze the process of activation without any supporting seeding. In what follows, we will call this process *basic*. As mentioned above, during primary seeding stage nodes in P are chosen as primary seeds. In first activation stage all nodes $p_{i,j}$, as well as all nodes $l_{i,j,1}$ get activated. In any subsequent activation stage t, such that $t > 1$ nodes that get activated are:

- nodes $l_{i,j,t}$ in activation stages where $t \leq T$;
- nodes $S_{t-1,j}$;
- nodes $s_{t-2,i,j}$ in activation stages where $t > 2$;
- nodes $a_{i,j,t-1-i}$ and $b_{i,j,t-1-i}$ in activation stages where $t > 2$.

Therefore, when the process ends, all nodes of the network are activated, with the exception of nodes in Z. Fig A presents nodes that get activated in each round in different colors.

In what follows, we will show that distribution ϕ^* is the only temporal distribution of supporting seeds that activates all nodes in the network.

Notice that since in activation stage t such that $t < T$ nodes $S_{t-1,j}$ get activated, then in supporting seeding stage t (that takes place after activation stage t) m inactive nodes with highest degrees are nodes $S_{t,j}$, with nodes where $j \leq \phi^*(t)$ having higher degree than these where $j > \phi^*(t)$. Therefore, activating any supporting seeds in stage t such that $t < T$ by some distribution ϕ will first activate any nodes $S_{t,j}$ such that $j \leq \phi^*(t)$ and then any remaining nodes $S_{t,j}$ such that $j > \phi^*(t)$. In the last supporting seeding stage (so when $t = T$) inactive nodes with highest degrees are nodes $S_{t,j}$ where $j \leq \phi^*(t)$. Therefore, if there are any such nodes exist, *i.e.*, if $\phi^*(t) > 0$), they will be chosen as supporting seeds first. Next candidates for supporting seeds are inactive nodes $a_{i,j,T-1}$ and $b_{i,j,T-1}$ with degrees $x + 1$ (if there are any such inactive nodes) and finally nodes $z_{i,j,k}$ and $S_{t,j}$ where $j > \phi^*(t)$ with degree 1.

Notice also that selecting $S_{i,j}$ as a supporting seed causes all nodes in the subtree of $S_{i,j}$ that get activated in the basic process (so nodes $s_{i,j,k}$, $a_{i,j,k}$ and $b_{i,j,k}$) to get activated one activation stage earlier than in the basic process. This is because in activation stage $i + 1$, when in the basic process $S_{i,j}$ would be activated, now its inactive neighbours get activated (that normally would be activated in activation stage $i + 2$).

Their neighbours get activated in activation stage $i + 2$ (instead of activation stage $i + 3$), *etc.*. Therefore, the nodes $z_{i,j,k}$ that do not get activated at all in the basic process, get activated in the last activation stage $T + 1$ if node $S_{i,j}$ is chosen as supporting seed.

Hence, one way of activating all nodes $z_{i,j,k}$ (and, consequently, entire network) is choosing a distribution of supporting seeds that chooses as supporting seeds all nodes $S_{i,j}$ that have nodes $z_{i,j,k}$ in their subtrees. Because of the way in which we constructed network G these are exactly nodes $S_{i,j}$ such that $j \leq \phi^*(i)$. As showed above, selecting $\phi^*(i)$ supporting seeds in supporting seeding stage i will activate exactly these nodes $S_{i,j}$ such that $j \leq \phi^*(i)$ (because they are inactive nodes with highest degrees). Therefore, choosing ϕ^* as the distribution of supporting seeds activates entire network.

We will now show that any different distribution ϕ of supporting seeds leaves some of the nodes inactive. Since $\phi \neq \phi^*$, at least one node $S_{i,j}$ such that $j \leq \phi^*(i)$ is not chosen as supporting seed (because there are exactly m such nodes). We will show that it is impossible to activate all nodes $z_{i,j,k}$ in the subtree of $S_{i,j}$ using distribution $\phi \neq \phi^*$. Notice that choosing supporting seed in any other subtree than this of node $S_{i,j}$ does not affect activation in the subtree of $S_{i,j}$. Therefore, only possible choices of supporting seeds that can affect activation of $z_{i,j,k}$ are nodes $a_{i,j,T-1}$, $b_{i,j,T-1}$, and $z_{i,j,k}$ that, as showed above, can be chosen as supporting seeds in supporting seeding stage T. However, to activate all nodes $z_{i,j,k}$ we would need to choose both $a_{i,j,T-1}$ and $b_{i,j,T-1}$ as supporting seeds. Choosing both $a_{i,j,T-1}$ and $b_{i,j,T-1}$ as supporting seeds would cause us to not choose some other node $S_{i',j'}$, which in turn would cause nodes $z_{i',j',k}$ to remain inactive. Hence, not choosing any $S_{i,j}$ such that $j \leq \phi^*(i)$ as supporting seed would cause at least x nodes $z_{i,j,k}$ to remain inactive (these are the nodes connected to the one of the nodes $a_{i,j,T-1}$ and $b_{i,j,T-1}$ that is not chosen). Hence, distribution ϕ^* is the only distribution that ensures activation of the entire network.

Finally, notice the since the difference in the number of activated nodes between ϕ^* and any other distribution is at least x, it can be arbitrary high, as the only limitation we put on x is that $x > m$. This concludes the proof.

Proof of Theorem 2

The decision version of the optimization problem is the following: given a network $G = (V, E)$, a number of primary seeds p, a number of supporting seeds m, a number of supporting seeding stages T, a probability of activation PP, a strategy of choosing seeds SR, and a value $r^* \in \mathbb{R}$, does there exist a temporal distribution of the supporting seeds ϕ such that the expected number of activated nodes is at least r^*.

The main idea of the NP-hardness proof is as follows. We will show a reduction from the NP-complete 3-Set Cover problem. We build a network that reflects the structure of a given 3-Set Cover problem instance and use it as an input for the Supporting Seeding problem. Finally, we show that an optimal solution of the Supporting Seeding problem corresponds to a solution of the given instance of the 3-Set Cover problem.

An instance of the NP-complete 3-Set Cover problem is defined by a universe $U = \{u_1, \ldots, u_{|U|}\}$, a collection of sets $S = \{S_1, \ldots, S_{|S|}\}$ such that $\forall_i S_i \subset U$ and $\forall_i |S_i| = 3$, and an integer $b \leq |S|$. The goal is to determine whether there exist b elements of S the union of which equals U. We will also assume that $|U| > b$. If this is not the case then the problem is trivial, as it is enough to pick for every u_j one set S_i such that $u_j \in S_i$. In what follows, let $\sigma(u_i) = |\{S_j \in S : u_i \in S_j\}|$, *i.e.*, $\sigma(u_i)$ is the number of sets in S that contain u_i.

First, let us create a network G, reflecting the structure of the given instance of the 3-Set Cover problem, as shown in Fig B:

- **The set of nodes:**

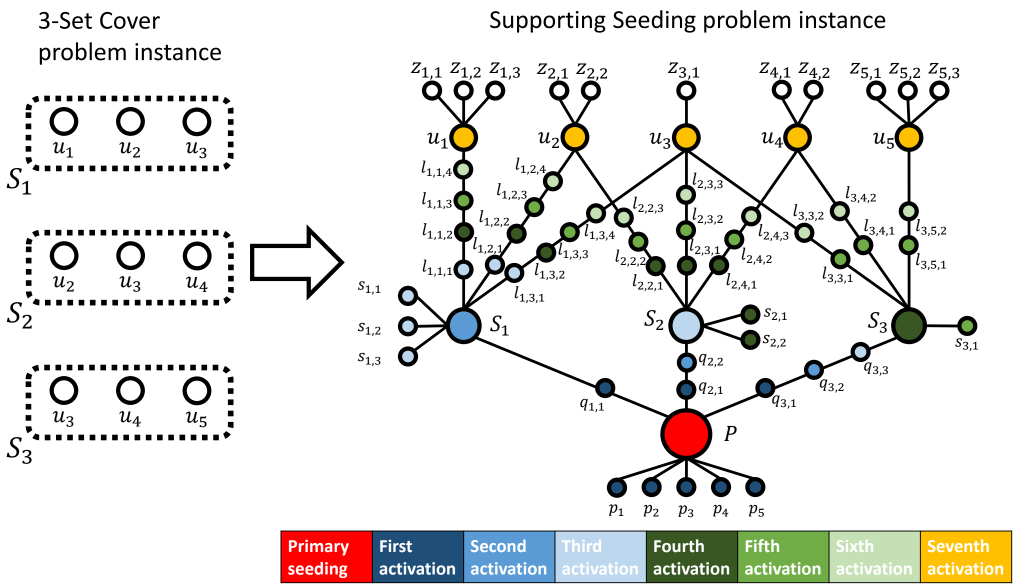

Fig B. Example of construction from the NP-hardness proof of the Supporting Seeding problem. Nodes that get activated in a process without any supporting seeding are colored according to their activation stage. Size of the node corresponds to its degree.

- For every $S_i \in S$, we create a single node, denoted by S_i, as well as $2|S| - 2 - i$ nodes $s_{i,j}, \ldots, s_{i,2|S|-2-i}$ and i nodes $q_{i,1}, \ldots, q_{i,i}$.
- For every $u_i \in U$, we create a single node, denoted by u_i, as well as $|S| + 1 - \sigma(u_i)$ nodes $z_{i,1}, \ldots, z_{i,|S|+1-\sigma(u_i)}$.
- For every $u_j \in U$ and every $S_i \in S$ such that $u_j \in S_i$ we create $2|S| - 1 - i$ nodes $l_{i,j,1}, \ldots, l_{i,j,2|S|-1-i}$.
- Additionally, we create a single node P and $|S| + 2$ nodes $p_1, \ldots, p_{|S|+2}$.

- **The set of edges:**
  - For every node p_i we create an edge (p_i, P).
  - For every node $q_{i,1}$ we create an edge $(q_{i,1}, P)$.
  - For every pair of nodes $q_{i,j}$, $q_{i,j+1}$ we create an edge $(q_{i,j}, q_{i,j+1})$.
  - For every node $q_{i,i}$ we create an edge $(q_{i,i}, S_i)$.
  - For every node $s_{i,j}$ we create an edge $(s_{i,j}, S_i)$.
  - For every node $l_{i,j,1}$ we create an edge $(l_{i,j,1}, S_i)$.
  - For every pair of nodes $l_{i,j,k}$, $l_{i,j,k+1}$ we create an edge $(l_{i,j,k}, l_{i,j,k+1})$.
  - For every node $l_{i,j,2|S|-1-i}$ we create an edge $(l_{i,j,2|S|-1-i}, u_j)$.
  - For every node $z_{i,j}$ we create an edge $(z_{i,j}, u_i)$.

Let the number of primary seeds be $p = 1$, the number of supporting seeds $m = b$ (where b is the parameter of the 3-Set Cover problem instance, *i.e.*, the maximal size of cover), the number of supporting seeding stages $T = 2|S|$, and the probability of activation $PP = 1$. Also, let SR be the strategy of choosing supporting seeds according to their degree, and let $r^* = |V|$, *i.e.*, we want to activate all nodes in the network.

Now, consider the instance of problem of Supporting Seeding in the form of $(G, p, m, T, PP, SR, r^*)$. We will now show that an optimal solution to this instance corresponds to an optimal solution to the given instance of the 3-Set Cover problem.

First, let us analyze the degrees of nodes in V as, due to the chosen strategy of seeding, they determine which nodes become seeds:

- We have $d(P) = 2|S| + 2$, because it is connected to $|S|$ nodes $q_{i,1}$, as well as to $|S| + 2$ nodes p_i.
- We have $d(S_i) = 2|S| + 2 - i$ because it is connected to $2|S| - 2 - i$ nodes $s_{i,j}$, to node $q_{i,i}$, as well as to 3 nodes $l_{i,j,1}$. Notice that this implies that for any node S_i we have $|S| + 2 \leq d(S_i) \leq 2|S| + 1$.
- We have $d(u_j) = |S| + 1$ because it is connected to $\sigma(u_j)$ nodes $l_{i,j,2|S|-1-i}$, as well as to $|S| + 1 - \sigma(u_j)$ nodes $z_{j,i}$.
- Finally, for all other nodes $v \in V$ we have $d(v) \leq 2$.

Now, let us analyze the process of activation without any supporting seeding. In what follows, we will call this process *basic*. Since node P has the highest degree in the network, it will be chosen as the primary seed. Since we have probability of activation $PP = 1$, the activation stage in which node gets activated is deterministic, and depends solely on the distance between said node and the primary seed.

In first activation stage all nodes p_i, as well as all nodes $q_{i,1}$ get activated. In any subsequent activation stage t, such that $t > 1$ nodes that get activated are:

- nodes $q_{i,t}$ in activation stages where $t \leq |S|$;
- node S_{t-1} in activation stages where $2 \leq t \leq |S| + 1$;
- nodes $s_{t-2,i}$ in activation stages where $3 \leq t \leq |S| + 2$;
- nodes $l_{i,j,t-1-i}$ in activation stages where $3 \leq t \leq 2|S|$;
- nodes u_i in activation stage $t = 2|S| + 1$.

Therefore, when the process ends, all nodes of the network are activated, with the exception of nodes $z_{i,j}$. Fig B presents nodes that get activated in each round in different colors.

Notice that activating x supporting seeds in supporting seeding stage t will activate up to x not yet activated nodes S_i with highest degrees (if there still are any such nodes), and possibly some of the nodes u_i (they all have the same degree) if there are not enough inactive nodes S_i. This is because these are the nodes with highest degrees that can be used as supporting seeds (as the node P, with the highest degree in the network, is activated as the primary seed). Since nodes u_i are activated in the basic process during the very last activation stage, and since we assumed that $|U| > b$, there are always enough inactive u_i nodes to be selected as supporting seeds. Therefore, every possible distribution of supporting seeds corresponds to activating a subset of nodes S_i and nodes u_i earlier than in the basic process.

Let us now analyze how choosing either node S_i or node u_i as a supporting seed leads to activating some of the nodes $z_{j,k}$. Earlier activation of any node S_i chosen as a supporting seed leads to earlier activation of all nodes $l_{i,j,k}$, in comparison to the basic process. This leads to earlier activation of all nodes u_j such that $u_j \in S_i$, which results in activating nodes $z_{j,k}$, that are not activated at all in the basic process. Notice also that choosing S_i as a supporting seed does not lead to earlier activation of any other node S_j, as any path leading from S_i to S_j through a node u_k in $S_i \cap S_j$ is longer than

$T = 2|S|$, while the last node S_j is activated in the basic process in stage $|S|+1$. This means that choosing S_i as a supporting seed does not change activation stage of any node $u_j \notin S_i$ and, consequently, do not cause activation of any other nodes $z_{j,k}$ where $u_j \notin S_i$. Alternatively, choosing one of the nodes u_i as a supporting seed leads to activation of all nodes $z_{i,j}$, which are not activated in the basic process. Hence, to activate all nodes $z_{j,k}$ (which is necessary to get the expected number of activated node equal to $|V|$), we have to earlier activate all nodes u_j, either by choosing them as supporting seeds, or by choosing as supporting seed at least one S_i such that $u_j \in S_i$.

To prove NP-hardness of the Supporting Seeding problem we will now show that a solution to the given instance of the 3-Set Cover problem exists if and only if there exists a solution to the constructed instance of the Supporting Seeding problem.

First, we will show that existence of a solution S^* to the given instance of the 3-Set Cover problem implies existence of a solution to the constructed instance of the Supporting Seeding problem. Indeed, such solution can be created by activating one supporting seed in each round i such that $S_i \in S^*$ (for such distribution supporting seeds will be exactly all nodes S_i such that $S_i \in S^*$, as in round i node S_i has the highest degree among all inactive nodes in the network). Since S^* is a solution to the given instance of the 3-Set Cover problem, then for every u_j there exists S_i such that $u_j \in S_i$ and S_i gets activated earlier than in the basic process. Hence, based on analysis provided in the previous paragraphs, every u_j also gets activated earlier than in the basic process, which leads to activating all nodes $z_{j,k}$ and getting the expected number of activated nodes equal to $|V|$.

Finally, we will show that existence of a solution to the constructed instance of the Supporting Seeding problem, *i.e.*, distribution ϕ such that the expected number of activated nodes is $|V|$, implies existence of a solution to the given instance of the 3-Set Cover problem. As described above, every such distribution ϕ leads to activating some of the nodes S_i and u_j as supporting seeds. Since the ϕ is solution to the constructed instance of the Supporting Seeding problem, all nodes u_j are activated earlier than in the basic process (as already discussed, it is necessary to activate all nodes $z_{j,k}$). Therefore, every node u_j is either chosen as supporting seed, or there exists at least one S_i such that $u_j \in S_i$ and S_i is chosen as supporting seed. Hence, we can construct a solution to the given instance of the 3-Set Cover problem by selecting every S_i that was chosen as a supporting seed, and by selecting one S_i such that $u_j \in S_i$ for every u_j that was chosen as a supporting seed (if such S_i does not exists, *i.e.*, there is no S_i such that $u_j \in S_i$, then clearly the given instance does not have a solution). Since the number of supporting seeds is at most b, such constructed cover is also of size at most b and it is a valid solution to the given instance of the 3-Set Cover problem.

This concludes the proof.

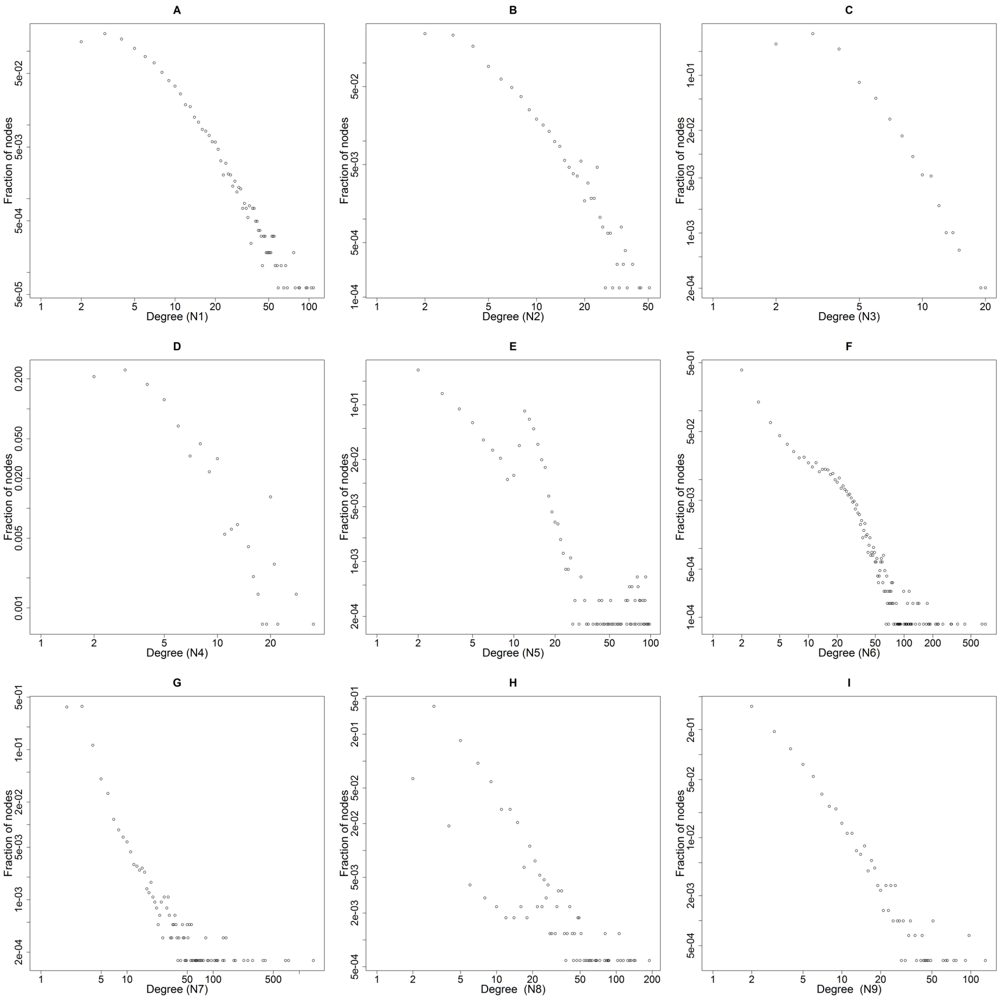

Fig C. Degree distributions for real networks N1-N9 (C1) Degree distribution for network N1; (C2) Degree distribution for network N2; (C3) Degree distribution for network N3; (C4) Degree distribution for network N4; (C5) Degree distribution for network N5; (C6) Degree distribution for network N6; (C7) Degree distribution for network N7; (C8) Degree distribution for network N8; (C9) Degree distribution for network N9

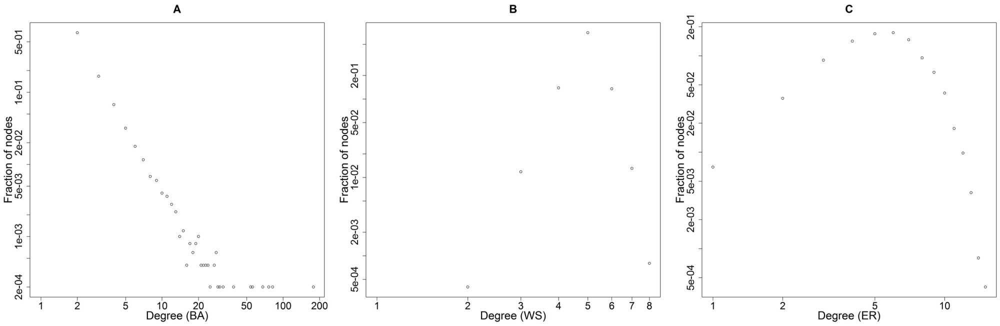

Fig D. Degree distributions for synthetic networks (D1) Degree distribution for synthetic network based on BA model; (D2) Degree distribution for synthetic network based on WS model; (D3) Degree distribution for network synthetic based on ER model

Simulation results for random networks

Table A. Average results for synthetic networks based on BA model

Parameter	Value	Average results				p-value			
		**Linear**	**GeomA**	**GeomD**	**Gaussian**	**Linear**	**GeomA**	**GeomD**	**Gaussian**
**PP**	0.05	3.31	3.69	3.69	3.65	$< 2e^{-16}$	$< 2e^{-16}$	$< 2e^{-16}$	$< 2e^{-16}$
	0.1	2.94	3.40	3.39	3.31	$< 2e^{-16}$	$< 2e^{-16}$	$< 2e^{-16}$	$< 2e^{-16}$
	0.15	2.77	2.87	2.80	2.81	$< 2e^{-16}$	$< 2e^{-16}$	$< 2e^{-16}$	$< 2e^{-16}$
	0.2	1.49	1.56	1.54	1.55	$< 2e^{-16}$	$< 2e^{-16}$	$< 2e^{-16}$	$< 2e^{-16}$
	0.25	1.24	1.28	1.25	1.28	$< 2e^{-16}$	$< 2e^{-16}$	$< 2e^{-16}$	$< 2e^{-16}$
	0.3	1.14	1.17	1.14	1.16	$< 2e^{-16}$	$< 2e^{-16}$	$< 2e^{-16}$	$< 2e^{-16}$
	0.35	1.10	1.12	1.09	1.11	$< 2e^{-16}$	$< 2e^{-16}$	$< 2e^{-16}$	$< 2e^{-16}$
	0.4	1.07	1.09	1.07	1.08	$< 2e^{-16}$	$< 2e^{-16}$	$< 2e^{-16}$	$< 2e^{-16}$
	0.45	1.06	1.07	1.05	1.06	$< 2e^{-16}$	$< 2e^{-16}$	$2.34e^{-16}$	$< 2e^{-16}$
	0.5	1.04	1.06	1.04	1.05	$4.41e^{-16}$	$< 2e^{-16}$	$4.41e^{-16}$	$2.83e^{-16}$
	0.55	1.04	1.05	1.04	1.04	$6.84e^{-16}$	$2.34e^{-16}$	$7.06e^{-16}$	$5.00e^{-16}$
	0.6	1.03	1.04	1.03	1.03	$1.06e^{-15}$	$4.01e^{-16}$	$1.03e^{-15}$	$8.51e^{-16}$
	0.65	1.03	1.04	1.03	1.03	$1.24e^{-15}$	$5.67e^{-16}$	$1.24e^{-15}$	$1.24e^{-15}$
	0.7	1.02	1.03	1.02	1.02	$1.58e^{-15}$	$8.78e^{-16}$	$1.49e^{-15}$	$1.63e^{-15}$
	0.75	1.02	1.03	1.02	1.02	$1.91e^{-15}$	$1.09e^{-15}$	$1.79e^{-15}$	$1.91e^{-15}$
	0.8	1.01	1.02	1.02	1.01	$2.59e^{-15}$	$2.03e^{-15}$	$2.29e^{-15}$	$3.11e^{-15}$
	0.85	1.01	1.01	1.01	1.01	$3.63e^{-15}$	$3.31e^{-15}$	$3.31e^{-15}$	$4.23e^{-15}$
	0.9	1.00	1.00	1.00	1.00	$4.77e^{-15}$	$4.63e^{-15}$	$4.77e^{-15}$	$5.39e^{-15}$
	0.95	1.00	1.00	1.00	1.00	$6.09e^{-15}$	$6.09e^{-15}$	$6.09e^{-15}$	$6.09e^{-15}$
	1	1.00	1.00	1.00	1.00	$6.09e^{-15}$	$6.09e^{-15}$	$6.09e^{-15}$	$6.09e^{-15}$
**Strategy**	D	1.27	1.28	1.27	1.27	$< 2e^{-16}$	$< 2e^{-16}$	$< 2e^{-16}$	$< 2e^{-16}$
	PR	1.57	1.58	1.58	1.57	$< 2e^{-16}$	$< 2e^{-16}$	$< 2e^{-16}$	$< 2e^{-16}$
	EV	1.38	1.52	1.49	1.49	$< 2e^{-16}$	$< 2e^{-16}$	$< 2e^{-16}$	$< 2e^{-16}$
	BT	1.24	1.33	1.31	1.31	$< 2e^{-16}$	$< 2e^{-16}$	$< 2e^{-16}$	$< 2e^{-16}$
**Support**	100%	1.14	1.19	1.18	1.18	$< 2e^{-16}$	$< 2e^{-16}$	$< 2e^{-16}$	$< 2e^{-16}$
	200%	1.26	1.32	1.30	1.30	$< 2e^{-16}$	$< 2e^{-16}$	$< 2e^{-16}$	$< 2e^{-16}$
	300%	1.37	1.43	1.42	1.42	$< 2e^{-16}$	$< 2e^{-16}$	$< 2e^{-16}$	$< 2e^{-16}$
	400%	1.48	1.55	1.53	1.53	$< 2e^{-16}$	$< 2e^{-16}$	$< 2e^{-16}$	$< 2e^{-16}$
	500%	1.58	1.65	1.63	1.63	$< 2e^{-16}$	$< 2e^{-16}$	$< 2e^{-16}$	$< 2e^{-16}$

Table B. Average results for synthetic networks based on WS model

Parameter	Value	Average results				p-value			
		**Linear**	**GeomA**	**GeomD**	**Gaussian**	**Linear**	**GeomA**	**GeomD**	**Gaussian**
**PP**	0.05	3.92	3.89	3.89	3.87	$< 2e^{-16}$	$< 2e^{-16}$	$< 2e^{-16}$	$< 2e^{-16}$
	0.10	3.87	3.80	3.85	3.79	$< 2e^{-16}$	$< 2e^{-16}$	$< 2e^{-16}$	$< 2e^{-16}$
	0.15	3.84	3.76	3.85	3.68	$< 2e^{-16}$	$< 2e^{-16}$	$< 2e^{-16}$	$< 2e^{-16}$
	0.20	3.83	3.68	3.79	3.56	$< 2e^{-16}$	$< 2e^{-16}$	$< 2e^{-16}$	$< 2e^{-16}$
	0.25	3.80	3.54	3.73	3.51	$< 2e^{-16}$	$< 2e^{-16}$	$< 2e^{-16}$	$< 2e^{-16}$
	0.30	3.69	3.51	3.63	3.43	$< 2e^{-16}$	$< 2e^{-16}$	$< 2e^{-16}$	$< 2e^{-16}$
	0.35	3.41	3.22	3.35	3.23	$< 2e^{-16}$	$< 2e^{-16}$	$< 2e^{-16}$	$< 2e^{-16}$
	0.40	2.60	2.52	2.52	2.55	$< 2e^{-16}$	$< 2e^{-16}$	$< 2e^{-16}$	$< 2e^{-16}$
	0.45	1.64	1.63	1.57	1.63	$2.49e^{-16}$	$5.36e^{-16}$	$4.36e^{-15}$	$5.26e^{-16}$
	0.50	1.25	1.24	1.21	1.23	$4.34e^{-10}$	$5.33e^{-10}$	$1.40e^{-09}$	$7.64e^{-10}$
	0.55	1.12	1.12	1.10	1.11	$2.12e^{-08}$	$1.86e^{-08}$	$3.48e^{-08}$	$2.73e^{-08}$
	0.60	1.07	1.08	1.06	1.06	$7.92e^{-08}$	$6.71e^{-08}$	$9.60e^{-08}$	$9.47e^{-08}$
	0.65	1.04	1.05	1.04	1.04	$1.77e^{-07}$	$1.44e^{-07}$	$1.81e^{-07}$	$2.07e^{-07}$
	0.70	1.02	1.03	1.02	1.02	$2.85e^{-07}$	$2.50e^{-07}$	$2.78e^{-07}$	$3.30e^{-07}$
	0.75	1.01	1.01	1.01	1.01	$4.02e^{-07}$	$4.02e^{-07}$	$4.02e^{-07}$	$4.29e^{-07}$
	0.80	1.00	1.00	1.00	1.00	$4.88e^{-07}$	$4.70e^{-07}$	$4.70e^{-07}$	$5.21e^{-07}$
	0.85	1.00	1.00	1.00	1.00	$5.21e^{-07}$	$5.21e^{-07}$	$5.21e^{-07}$	$5.21e^{-07}$
	0.90	1.00	1.00	1.00	1.00	$5.21e^{-07}$	$5.21e^{-07}$	$5.21e^{-07}$	$5.21e^{-07}$
	0.95	1.00	1.00	1.00	1.00	$5.21e^{-07}$	$5.21e^{-07}$	$5.21e^{-07}$	$5.21e^{-07}$
	1.00	1.00	1.00	1.00	1.00	$5.21e^{-07}$	$5.21e^{-07}$	$5.21e^{-07}$	$5.21e^{-07}$
**Strategy**	D	1.86	1.87	1.87	1.86	$< 2e^{-16}$	$< 2e^{-16}$	$< 2e^{-16}$	$< 2e^{-16}$
	PR	2.03	2.02	2.02	2.00	$< 2e^{-16}$	$< 2e^{-16}$	$< 2e^{-16}$	$< 2e^{-16}$
	EV	2.08	1.83	1.87	1.80	$< 2e^{-16}$	$< 2e^{-16}$	$< 2e^{-16}$	$< 2e^{-16}$
	BT	2.45	2.50	2.57	2.48	$< 2e^{-16}$	$< 2e^{-16}$	$< 2e^{-16}$	$< 2e^{-16}$
**Support**	100%	1.41	1.41	1.40	1.39	$< 2e^{-16}$	$< 2e^{-16}$	$< 2e^{-16}$	$< 2e^{-16}$
	200%	1.79	1.76	1.76	1.74	$< 2e^{-16}$	$< 2e^{-16}$	$< 2e^{-16}$	$< 2e^{-16}$
	300%	2.13	2.07	2.10	2.06	$< 2e^{-16}$	$< 2e^{-16}$	$< 2e^{-16}$	$< 2e^{-16}$
	400%	2.45	2.37	2.42	2.35	$< 2e^{-16}$	$< 2e^{-16}$	$< 2e^{-16}$	$< 2e^{-16}$
	500%	2.76	2.66	2.73	2.64	$< 2e^{-16}$	$< 2e^{-16}$	$< 2e^{-16}$	$< 2e^{-16}$

Table C. Average results for synthetic networks based on ER model

Parameter	Value	Average results				p-value			
		**Linear**	**GeomA**	**GeomD**	**Gaussian**	**Linear**	**GeomA**	**GeomD**	**Gaussian**
**PP**	0.05	3.78	3.89	3.88	3.85	$< 2e^{-16}$	$< 2e^{-16}$	$< 2e^{-16}$	$< 2e^{-16}$
	0.10	3.63	3.79	3.78	3.68	$< 2e^{-16}$	$< 2e^{-16}$	$< 2e^{-16}$	$< 2e^{-16}$
	0.15	3.06	3.24	3.31	3.26	$< 2e^{-16}$	$< 2e^{-16}$	$< 2e^{-16}$	$< 2e^{-16}$
	0.20	1.97	2.04	2.05	2.06	$< 2e^{-16}$	$< 2e^{-16}$	$< 2e^{-16}$	$< 2e^{-16}$
	0.25	1.24	1.27	1.23	1.25	$< 2e^{-16}$	$< 2e^{-16}$	$< 2e^{-16}$	$< 2e^{-16}$
	0.30	1.10	1.13	1.09	1.11	$< 2e^{-16}$	$< 2e^{-16}$	$< 2e^{-16}$	$< 2e^{-16}$
	0.35	1.06	1.08	1.05	1.06	$< 2e^{-16}$	$< 2e^{-16}$	$< 2e^{-16}$	$< 2e^{-16}$
	0.40	1.04	1.06	1.04	1.04	$< 2e^{-16}$	$< 2e^{-16}$	$< 2e^{-16}$	$< 2e^{-16}$
	0.45	1.03	1.05	1.03	1.03	$< 2e^{-16}$	$< 2e^{-16}$	$< 2e^{-16}$	$< 2e^{-16}$
	0.50	1.03	1.04	1.03	1.02	$< 2e^{-16}$	$< 2e^{-16}$	$< 2e^{-16}$	$< 2e^{-16}$
	0.55	1.02	1.04	1.02	1.02	$< 2e^{-16}$	$< 2e^{-16}$	$< 2e^{-16}$	$< 2e^{-16}$
	0.60	1.02	1.03	1.02	1.02	$< 2e^{-16}$	$< 2e^{-16}$	$< 2e^{-16}$	$< 2e^{-16}$
	0.65	1.01	1.03	1.02	1.01	$< 2e^{-16}$	$< 2e^{-16}$	$< 2e^{-16}$	$< 2e^{-16}$
	0.70	1.01	1.03	1.02	1.01	$< 2e^{-16}$	$< 2e^{-16}$	$< 2e^{-16}$	$< 2e^{-16}$
	0.75	1.01	1.02	1.02	1.01	$< 2e^{-16}$	$< 2e^{-16}$	$< 2e^{-16}$	$< 2e^{-16}$
	0.80	1.01	1.02	1.02	1.01	$< 2e^{-16}$	$< 2e^{-16}$	$< 2e^{-16}$	$< 2e^{-16}$
	0.85	1.01	1.02	1.02	1.01	$< 2e^{-16}$	$< 2e^{-16}$	$< 2e^{-16}$	$< 2e^{-16}$
	0.90	1.01	1.02	1.02	1.01	$< 2e^{-16}$	$< 2e^{-16}$	$< 2e^{-16}$	$< 2e^{-16}$
	0.95	1.01	1.01	1.01	1.01	$< 2e^{-16}$	$< 2e^{-16}$	$< 2e^{-16}$	$< 2e^{-16}$
	1.00	1.00	1.00	1.00	1.00	$< 2e^{-16}$	$< 2e^{-16}$	$< 2e^{-16}$	$< 2e^{-16}$
**Strategy**	D	1.42	1.43	1.42	1.41	$< 2e^{-16}$	$< 2e^{-16}$	$< 2e^{-16}$	$< 2e^{-16}$
	PR	1.47	1.48	1.47	1.46	$< 2e^{-16}$	$< 2e^{-16}$	$< 2e^{-16}$	$< 2e^{-16}$
	EV	1.50	1.66	1.65	1.62	$< 2e^{-16}$	$< 2e^{-16}$	$< 2e^{-16}$	$< 2e^{-16}$
	BT	1.43	1.40	1.39	1.39	$< 2e^{-16}$	$< 2e^{-16}$	$< 2e^{-16}$	$< 2e^{-16}$
**Support**	100%	1.17	1.19	1.19	1.18	$< 2e^{-16}$	$< 2e^{-16}$	$< 2e^{-16}$	$< 2e^{-16}$
	200%	1.32	1.36	1.34	1.34	$< 2e^{-16}$	$< 2e^{-16}$	$< 2e^{-16}$	$< 2e^{-16}$
	300%	1.46	1.50	1.49	1.48	$< 2e^{-16}$	$< 2e^{-16}$	$< 2e^{-16}$	$< 2e^{-16}$
	400%	1.60	1.64	1.63	1.61	$< 2e^{-16}$	$< 2e^{-16}$	$< 2e^{-16}$	$< 2e^{-16}$
	500%	1.72	1.76	1.76	1.74	$< 2e^{-16}$	$< 2e^{-16}$	$< 2e^{-16}$	$< 2e^{-16}$

Role of network characteristics for used distributions

We further analyse the randomly generated networks to study the effect of network characteristics on the performance of used distributions of supporting seeding. Results presented in Fig E-G show how the performance of supporting seeding is affected by network characteristics represented by typical network metrics like betweenness, closeness, average degree, eigenvector and the size of second order neighbourhood.

While using the lowest propagation probability PP=0.05 (first column of each figure) similar results were obtained for all values of used network characteristics. For higher propagation probabilities (PP=0.25 and PP=0.5, in second and third column of each figure) the impact of changing network characteristics on the total coverage is much more noticeable.

As for differences between distributions of supporting seeds, relative performance of each distribution does not seem to be dependent on characteristics values, *i.e.*, for every pair of distributions one of them is consistently better for all values of a given characteristic. Comparison of differences between results for used distributions of supporting seeds was performed with Wilcoxon signed rank at 95 percent confidence interval. It confirmed statistical significance of results with p $<= 3.83E-06$ for all pairwise comparisons of supporting seeding distributions within BA networks, p $<= 5.69E-06$ for ER networks and p $<= 6.82E-07$ for WS networks.

Interestingly, networks generated using all three models, namely Barabási–Albert (BA), Erdos-Renyi model (ER) and Watts-Strogatz (WS), show the same tendencies in regard to how the coverage depends on network characteristics, as it can be seen in Fig E-G. It suggests that the diffusion performance under supporting seeding depends more on low level characteristics of the network structure, rather than its global properties (such as scale-freeness or lack thereof).

Panels 1-3 of each figure illustrate how the coverage generally decreases with average betweenness. Higher betweenness suggests networks where fewer pairs of nodes have direct connection (in a clique, which is a perfect network in terms of spreading activation process, all nodes have betweenness 0). Panels 4-6 of each figure show that total coverage tends to grow with average closeness in the network. High mean closeness implies that the average distance between a pair of nodes is shorter, hence it is easier for activation process to reach higher number of nodes in fewer rounds. Panels 7-9 of each figure show how the coverage grows with the average degree of the network, as in the independent cascade model adding more edges results in more opportunities to spread the activation process to other nodes. Panels 10-12 of each figure show that the coverage tends to grow with the average eigenvector centrality value, as large values of eigenvector centrality are usually obtained by the members of dense network structures that favour quick diffusion. Finally, panels 13-15 of each figure present how the total coverage grows with the mean size of second order neighbourhood, *i.e.*, the number of nodes in distance 2 from the given node. Again, larger neighbourhoods result in more nodes that can be easily reached by the activation process.

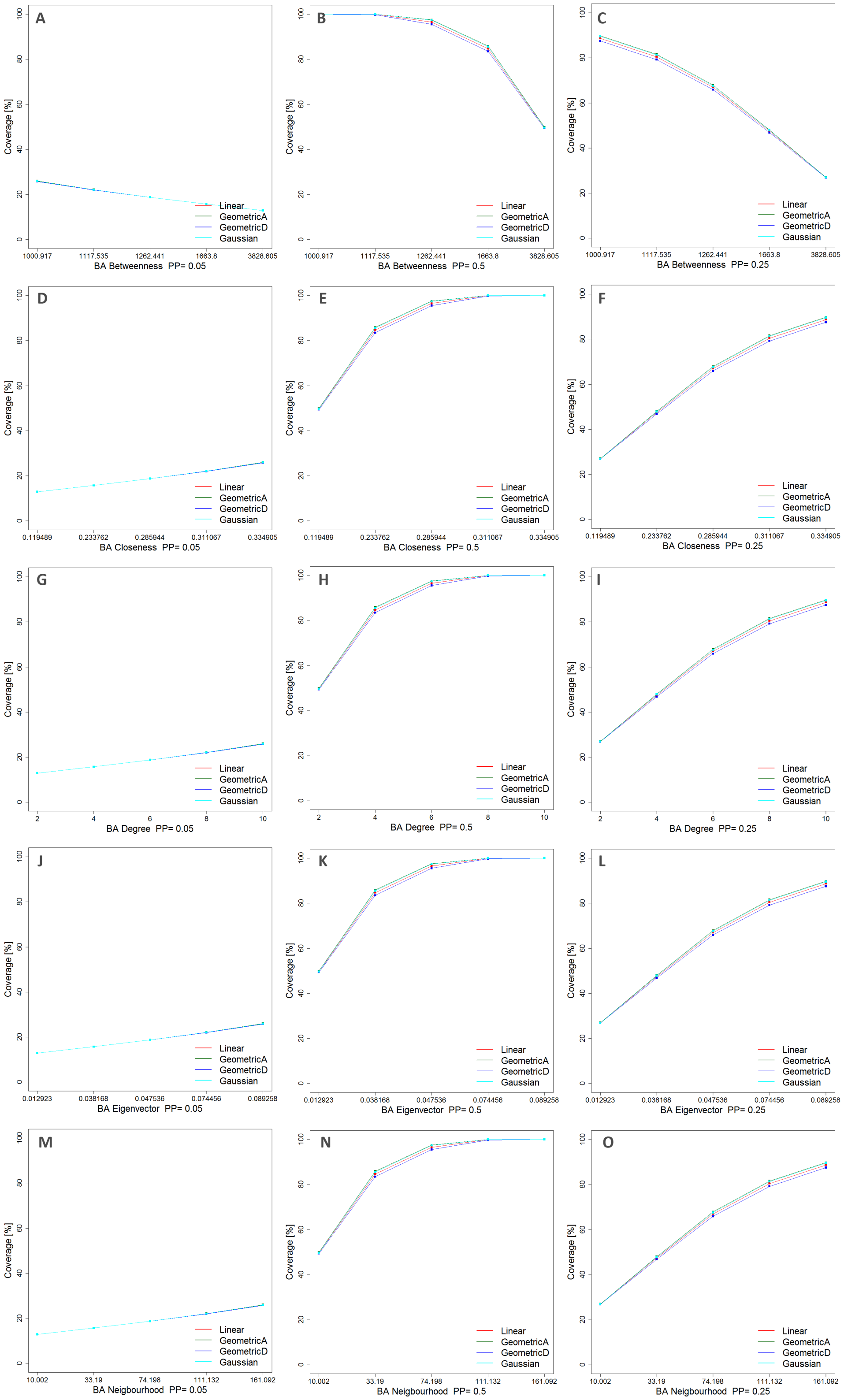

Fig E. Impact of network betweenness (E1,E2,E3), closeness (E4,E5,E6), degree (E7,E8,E9), eigenvector (E10,E11,E12) and neighbourhood (E13,E14,E15) on supporting seeding within BA networks for PP=0.05, PP=0.25 and PP=0.05

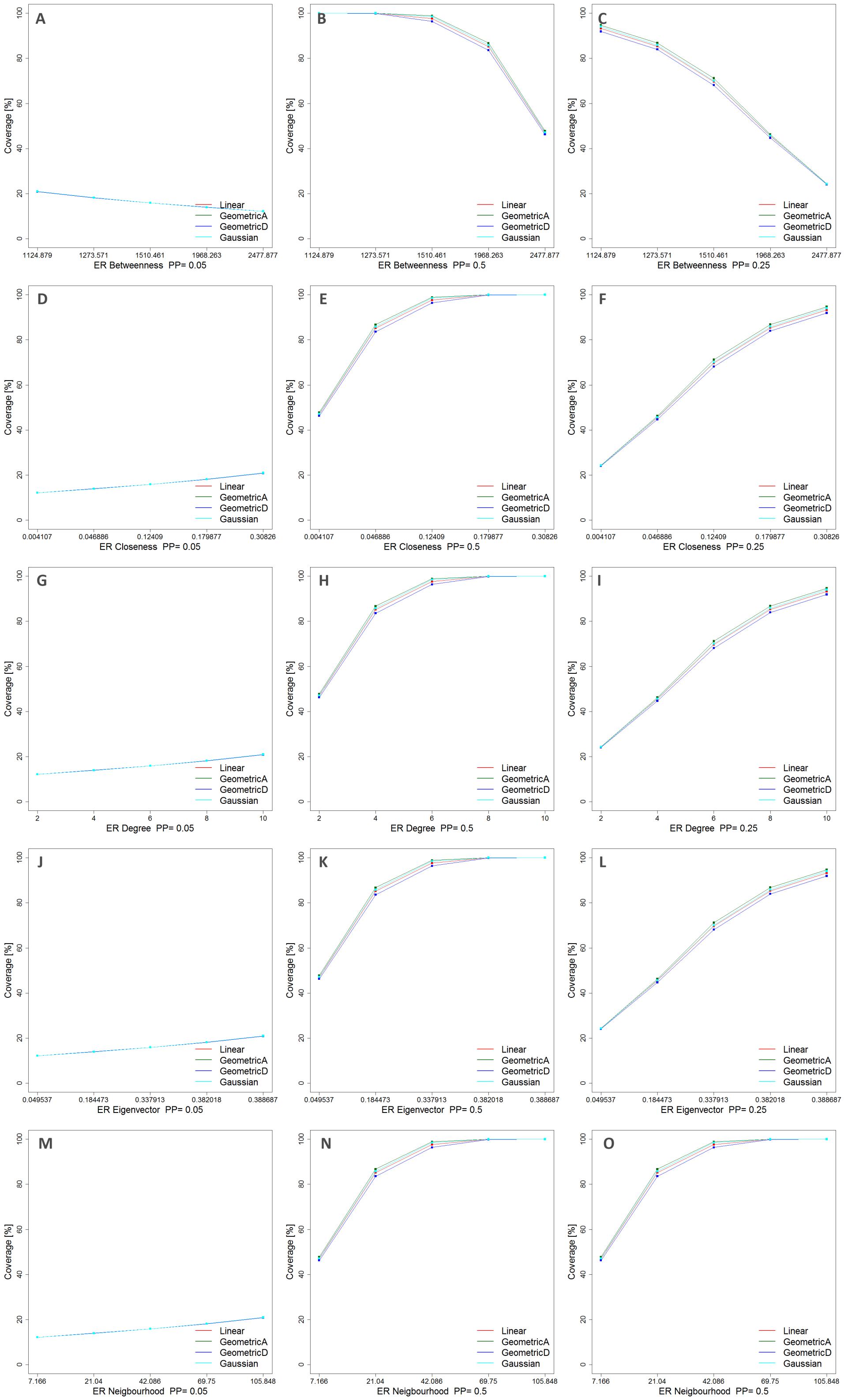

Fig F. Impact of network betweenness (F1,F2,F3), closeness (F4,F4,F6), degree (F7,F8,F9), eigenvector (F10,F11,F12) and neighbourhood (F13,F14,F15) on supporting seeding within ER networks for PP=0.05, PP=0.25 and PP=0.05

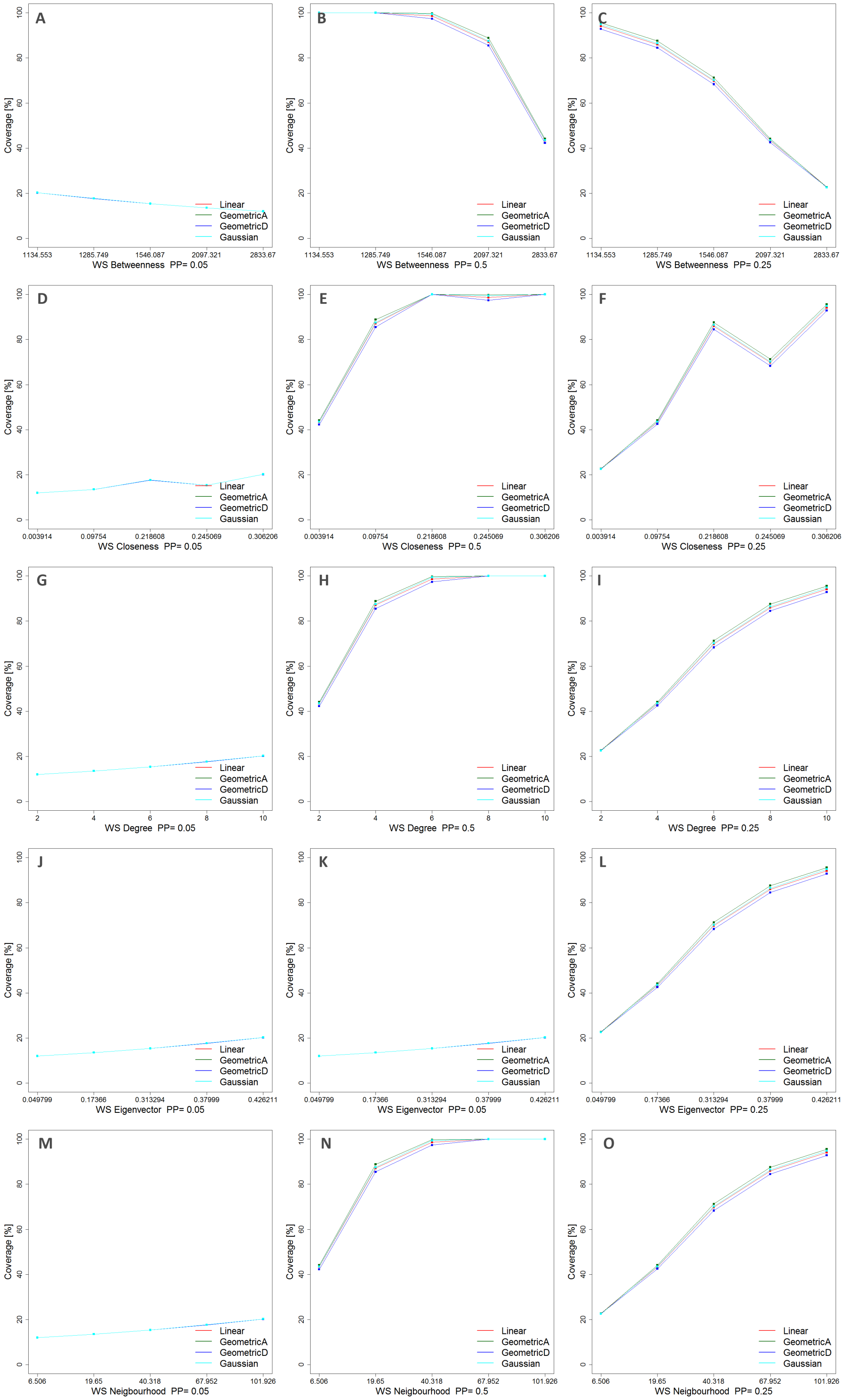

Fig G. Impact of network betweenness (G1,G2,G3), closeness (G4,G5,G6), degree (G7,G8,G9), eigenvector (G10,G11,G12) and neighbourhood (G13,G14,G15) on supporting seeding within WS networks for PP=0.05, PP=0.25 and PP=0.05
