# Supplementary material for: Strategic distribution of seeds to support diffusion in complex networks
Source: PLoS One. 2018 Oct 16;13(10):e0205130. doi: 10.1371/journal.pone.0205130 (PMC6191084; doi:10.1371/journal.pone.0205130)
Supplement: S1 Data — (ZIP) [file pone.0205130.s002.zip › Description.pdf]

# Description of used networks and included datasets

We include data sets necessary to replicate our study. It is divided into three parts.

## **Part 1** Real networks used for simulations

We used nine real networks from online data repositories. They are publicly available with references presented in Table 1.

| | |
|---|---|
| **network 1** | Newman MEJ. Scientific collaboration networks: I. Network construction and fundamental results. Phys. Rev. E 64, 016131, 2001. |
| **network 2** | Newman MEJ. The structure of scientific collaboration networks. Proc. Natl. Acad. Sci. USA 98, 404, 409, 2001. |
| **network 3** | Watts, DJ, Strogatz SH. Collective dynamics of `small, world' networks. Nature,393(1):440{442, 1998. |
| **network 4** | Newman MEJ. Finding community structure in networks using the eigenvectorsof matrices. Phys. Rev. E 74, 036104, 2006. |
| **network 5** | Leskovec J, Kleinberg J, Faloutsos C. Graph Evolution: Densification and Shrinking Diameters. ACM Transactions on Knowledge Discovery from Data (ACM TKDD), 1(1), 2007. |
| **network 6** | Ley M. The DBLP computer science bibliography: Evolution, research issues, perspectives. In String processing and information retrieval (pp. 481486). Springer Berlin/Heidelberg, 2002. |
| **network 7** | Leskovec J, Kleinberg J, Faloutsos C. Graph Evolution: Densification and Shrinking Diameters. ACM Transactions on Knowledge Discovery from Data (ACM TKDD), 1(1), 2007. |
| **network 8** | Stelzl U, Worm U, Lalowski M, Haenig C, Brembeck FH, Goehler H, et al. A human protein protein interaction network: a resource for annotating the proteome. Cell, 122(6), 957 968, 2005. |
| **network 9** | Rual JF, Venkatesan K, Hao T, Hirozane Kishikawa T. Dricot A, Li N, et al. Towards a proteome scale map of the human protein{protein interaction network. Nature, 437(7062), 11731178, 2005. |

Table 1. References of used real networks

Included data file **dataset_1.xls** localized in folder **Part1** contains detailed results from simulations within real networks. Description of variables from each column is presented in Table 2.

| | |
|---|---|
| **pp** | Propagation probability |
| **network** | ID of used network |
| **strategy** | Strategy of seed selection 1) Degree 2) PageRank 4) Eigenvector 5) Betweenness |
| **distribution** | Used distribution of seeds with 1) Linear distribution 2) Geometric ascending 3) Geometric descending 4) Gaussian |
| **sp** | Seeding percentage - percentage of nodes used as seeds |
| **support** | Number of seeds used as supporting seeds |
| **activations** | Number of activated nodes within network |
| **coverage** | Percentage of activated nodes |

Table 2. Description of columns from dataset with results from real networks

## Part 2 Synthetic networks used for simulations

Datasets used for identification of role of network models is based on  networks localized in folder **Part2**. They are generated with BA, ER and WS models.  Included data file dataset_2.xls localized in folder Part2 contains detailed results from simulations within synthetic networks. Description of variables from each column is presented in Table 2.

| | |
|---|---|
| **pp** | propagation probability |
| **model** | Information about theoretic model used for network generation (BA, ER, WS) |

| | |
|---|---|
| **network** | ID of used network |
| **strategy** | Strategy of seed selection 1) Degree 2) PageRank 4) Eigenvector 5) Betweenness |
| **distribution** | Used distribution of seeds with 1) Linear distribution 2) Geometric ascending 3) Geometric descending 4) Gaussian |
| **sp** | Seeding percentage - percentage of nodes used as seeds |
| **support** | Number of seeds used as supporting seeds |
| **activations** | Number of activated nodes within network |
| **coverage** | Percentage of activated nodes |

Table 3. Description of columns from data set file with results from real networks

## Part 3 Synthetic networks used for identification of role of network characteristics

Dataset used for identification of role of network parameters is based on networks localized in folder **Part3**. Networks 1-5 are generated according to BA model with average degree respectively 2,4,6,8 and 10. Networks 6-10 are generated according to ER model with average degree respectively 2,4,6,8 and 10. Networks 11-15 are generated according to WS model with average degree respectively 2,4,6,8 and 10 and rewiring probability 0.5.

Included data file dataset_3.xls localized in folder Part3 contains detailed results from simulations within synthetic network. Description of variables from each column is presented in Table 4.

| | |
|---|---|
| **pp** | propagation probability |
| **model** | Information about theoretic model used for network generation (BA, ER, WS) |
| **network** | ID of used networks BA model (1-5) ER model (6-10) WS model (11-15) |
| **dg** | average network degree |
| **ev** | average eigenvector |
| **bt** | average betweenness |
| **cl** | closeness |
| **nb** | average neighborhood |
| **distribution** | Used distribution of seeds with 1) Linear distribution 2) Geometric ascending 3) Geometric descending 4) Gaussian |
| **coverage** | Percentage of activated nodes |

Table 4. Description of columns from data set file with results from synthetic networks
